# Order *versus* disorder in two isomorphous pyrazolone-substituted diethyl propane­dioates prepared using a three-component one-pot reaction under solvent-free conditions

**DOI:** 10.1107/S2056989020011676

**Published:** 2020-09-08

**Authors:** Tharangini K. Shreekanth, Hemmige S. Yathirajan, Balakrishna Kalluraya, Sabine Foro, Christopher Glidewell

**Affiliations:** a Department of Studies in Chemistry, Mangalore University, Mangalagangotri, Mangalore-574 199, India; b Department of Studies in Chemistry, University of Mysore, Manasagangotri, Mysuru-570 006, India; c Institute of Materials Science, Darmstadt University of Technology, Alarich-Weiss-Strasse 2, D-64287 Darmstadt, Germany; d School of Chemistry, University of St Andrews, St Andrews, Fife KY16 9ST, UK

**Keywords:** synthesis, pyrazoles, crystal structure, disorder, mol­ecular conformation, hydrogen bonding, supra­molecular assembly

## Abstract

Two new substituted propane­dioate esters have been synthesized using a three-component solvent-free thermal reaction between diethyl propane­dioate (diethyl malonate), 5-chloro-3-methyl-1-phenyl-1*H*-pyrazole-4-carbaldehyde and an aryl azide, forming two new C—C bonds in a single step. The products diethyl (*RS*)-2-[(4-bromo­phen­yl)(5-methyl-3-oxo-2-phenyl-2,3-di­hydro-1*H*-pyrazol-4-yl)meth­yl]propane­dioate, C_24_H_25_BrN_2_O_5_ (I), and diethyl (*RS*)-2-[(4-chloro­phen­yl)(5-methyl-3-oxo-2-phenyl-2,3-di­hydro-1*H*-pyrazol-4-yl)meth­yl]propane­dioate, C_24_H_25_ClN_2_O_5_ (II), are isomorphous, with *Z*′ = 2 in space group *P*2_1_/*n*. The two independent mol­ecules in compound (I) are both fully ordered, while each of the independent mol­ecules in compound (II) is disordered, but in different ways. In one mol­ecule of (II), the *N*-phenyl ring is disordered over two sets of atomic sites having occupancies 0.635 (10) and 0.365 (10), and in the other mol­ecule the ester function is disordered over two sets of atomic sites having occupancies 0.690 (5) and 0.310 (5). In both structures, the two independent mol­ecules adopt different conformations and, in each structure, the mol­ecules are linked into complex sheets by a combination of N—H⋯O, C—H⋯O and C—H⋯π(arene) hydrogen bonds. Comparisons are made with some related structures.

## Abstract 2

Two new substituted propane­dioate esters have been synthesized using a three-component solvent-free thermal reaction. The products, which are isomorphous, differ only in the presence of a bromo­phenyl group in one, where the mol­ecules are fully ordered, as compared with a chloro­phenyl group in the other, where the mol­ecules exhibit two types of disorder.

## Chemical context   

**Figure 1:**
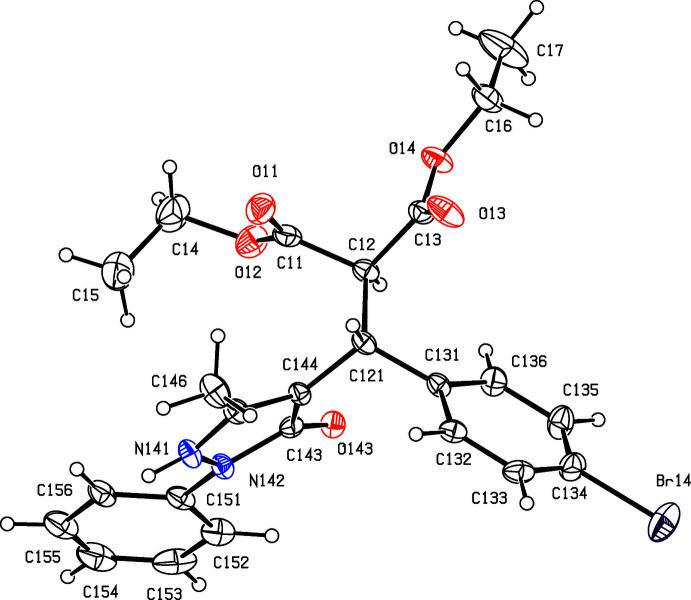
The structure of the type 1 mol­ecule of (I), showing the atom-labelling scheme. Displacement ellipsoids are drawn at the 30% probability level.

Pyrazoles exhibit a very wide range of pharmacological and other biological activities, which have recently been extensively reviewed (Ansari *et al.*, 2017 ▸; Karrouchi *et al.*, 2018 ▸). In a continuation of a broadly based study of the synthesis and structures of novel pyrazole derivatives (Asma *et al.*, 2018 ▸; Kiran Kumar *et al.*, 2020 ▸; Shaibah *et al.*, 2020*a* ▸,*b* ▸), we have now investigated a three-component reaction between di­ethyl­propane­dioate (di­ethyl­malonate), 5-chloro-3-methyl-1-phen­yl-1*H*-pyrazole-4-carbaldehyde and some aryl azides. Our expectation was that the methyl­ene group of the ester component would undergo a condensation reaction with the carbaldehyde function to provide a new electron-deficient alkene system, which would then undergo a 1,3-dipolar cyclo­addition with the aryl azide to provide pyrazole-substituted 1,2,3-triazoles. The reactions, carried out under thermal and solvent-free conditions, turned out to take an entirely different course, in which the azide group was lost and giving, instead of the anti­cipated products, the highly substituted esters diethyl (*RS*)-2-[(4-bromo­phen­yl)(5-methyl-3-oxo-2-phenyl-2,3-di­hydro-1*H*-pyrazol-4-yl)meth­yl]propane­dioate (I) (Figs. 1 ▸ and 2 ▸) and diethyl (*RS*)-2-[(4-chloro­phen­yl)(5-methyl-3-oxo-2-phenyl-2,3-di­hydro-1*H*-pyrazol-4-yl)meth­yl]propane­dioate (II) (Figs. 3 ▸ and 4 ▸). The yields were fairly low, in the range 35–40%, and the course of the reaction is unclear: the by-products must include HCl and HN_3_, and the H atoms in these by-products may well arise from thermal degradation of one or more of the reactants, particularly the ester component. However, despite the modest yields, compounds (I) and (II) are formed from readily accessible precursors in a very rapid process in which two new C—C bonds are formed in a single step. Here we report the synthesis of compounds (I) and (II), the reaction sequence for which is summarized in Fig. 5 ▸, and their mol­ecular and supra­molecular structures.
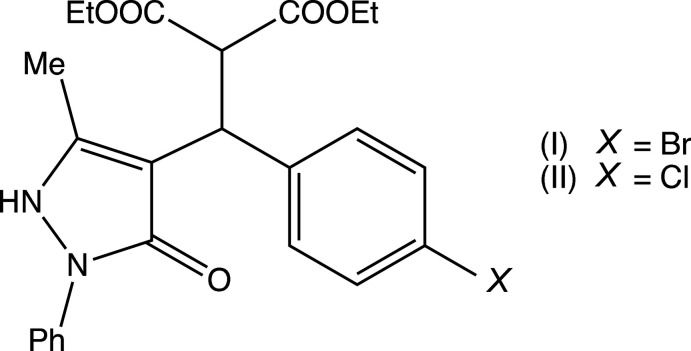


## Structural commentary   

Compounds (I) and (II) both crystallize with *Z*′ = 2 in space group P2_1_/*n*, and they are isomorphous. However, while the mol­ecules in (I) are both fully ordered (Figs. 1 ▸ and 2 ▸), albeit with some evidence for large librational motion in one of the eth­oxy groups, both of the independent mol­ecules exhibit disorder in (II). In the type 1 mol­ecule of (II), containing atom C121 (Fig. 3 ▸), the unsubstituted phenyl ring is disordered over two sets of atomic sites having occupancies 0.635 (10) and 0.365 (10), and in the type 2 mol­ecule, containing atom C221 (Fig. 4 ▸), the whole di­ethyl­malonate fragment is disordered over two sets of atomic sites having occupancies 0.690 (5) and 0.310 (5).

**Figure 2:**
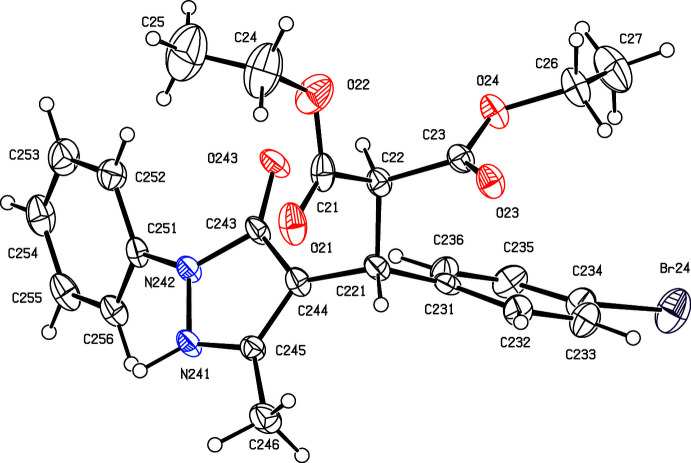
The structure of the type 2 mol­ecule of (I), showing the atom-labelling scheme. Displacement ellipsoids are drawn at the 30% probability level.

All of the mol­ecules contain a stereogenic centre, at atom C121 in the type 1 mol­ecules (Figs. 1 ▸ and 3 ▸) and at atom C221 in the type 2 mol­ecules (Figs. 2 ▸ and 4 ▸), and all of the reference mol­ecules were selected to have the *R*-configuration. The centrosymmetric space group confirms that both compounds have crystallized as racemic mixtures.

**Figure 3:**
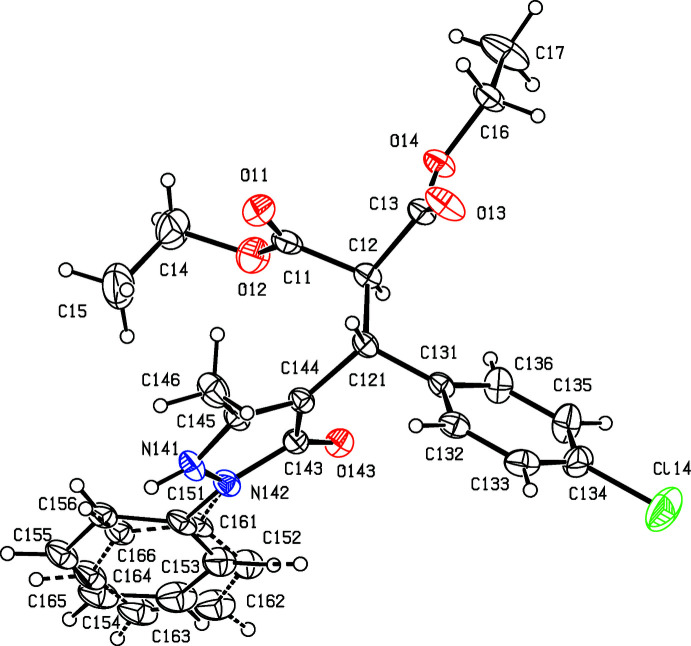
The structure of the type 1 mol­ecule of (II), showing the atom-labelling scheme and the disorder. The major disorder component is drawn using full lines and the minor disorder component is drawn using broken lines. Displacement ellipsoids are drawn at the 30% probability level.

In both mol­ecules of compound (I), the substituents on the C*x*2—C*x*21 bond (where *x* = 1 or 2; Figs. 1 ▸ and 2 ▸) adopt a conformation that is almost fully staggered, with the two H atoms anti­periplanar (Table 1 ▸); the same applies to compound (II) (Figs. 3 ▸ and 4 ▸), including both of the disorder components in the type 2 mol­ecule. However, comparison of other aspects of the mol­ecular conformations of the two independent mol­ecules in the ordered structure of (I) shows some marked differences between the two mol­ecules (Table 1 ▸; Figs. 1 ▸ and 2 ▸). In particular, the components of the diester function in the two mol­ecules are very different, as exemplified by the values of the torsional angles C*x*21—C*x*2—C*x*1—O*x*2 and C*x*21—C*x*2—C*x*3—O*x*4 (Figs. 1 ▸ and 2 ▸). Of the atoms in the eth­oxy groups, only atom C14 participates in the hydrogen bonding (Table 2 ▸); while this may influence the conformation of the eth­oxy group O12/C14/C15, the other eth­oxy groups are most probably adopting conformations that reflect their efficient accommodation in the spaces available in the supra­molecular assembly generated by the hydrogen bonds (*cf.* Section 3 below). Similar remarks apply to the conformations of the disordered ester units in compound (II), below. Similarly, the orientations of the aryl group in the two mol­ecules differ, as shown by the torsional angles C*x*2—C*x*21—C*x*31—C*x*32 and N*x*41—N*x*42—C*x*51—C*x*52 (where *x* = 1 or 2; Figs. 1 ▸ and 2 ▸). These differences may be associated with the different hydrogen-bonding behaviour of the two mol­ecules. Thus, different ester units in the two mol­ecules are involved in hydrogen bonding (Table 2 ▸). The aryl groups in both mol­ecules are involved in hydrogen bonding; the substituted ring provides donors in both mol­ecules, in a C—H⋯O hydrogen bond in the type 1 mol­ecule and in a C—H⋯π(arene) hydrogen bond in the type 2 mol­ecule, but only in the type 1 mol­ecule does the unsubstituted aryl ring act as a hydrogen-bond acceptor.

**Figure 4:**
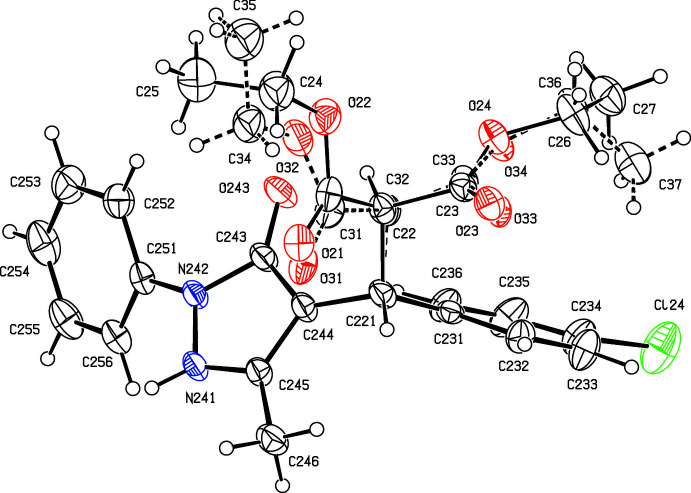
The structure of the type 2 mol­ecule of (II), showing the atom-labelling scheme and the disorder. The major disorder component is drawn using full lines and the minor disorder component is drawn using broken lines. Displacement ellipsoids are drawn at the 30% probability level and, for the sake of clarity, a few of the atom labels have been omitted.

In compound (II), the conformations of the major disorder components are very similar to those of the corresponding mol­ecules of compound (I), but those of the minor disorder components in the type 2 mol­ecule differ significantly (Table 1 ▸; Fig. 4 ▸), but the conformations of the two disorder components in the type 1 mol­ecule of (II) differ only modestly (Table 2 ▸; Fig. 3 ▸).

In compound (II) there is a rather short H⋯H contact, 1.79 Å, between the minor occupancy atom H163 in the reference mol­ecule 1 at (*x*, *y*, *z*) and the idealized riding site of the major occupancy atom H25*A* in mol­ecule 2 at (1 + *x*, *y*, *z*). However, the atom H25*A* forms part of a methyl group, and such methyl groups are likely to be undergoing extremely rapid rotations about the adjacent C—C bonds, particularly at ambient temperature (Riddell & Rogerson, 1996 ▸, 1997 ▸). Nonetheless, avoidance of this short contact distance would suggest that if the minor-occupancy form of mol­ecule 1 is present at (*x*, *y*, *z*), then mol­ecule 2 at (1 + *x*, *y*, *z*) will probably also be the minor-occupancy form. However, this does not imply any longer-range correlation between the disorder components, nor require any relationship between the disorder occupancy factors for the two independent mol­ecules.

Compounds (I) and (II) were crystallized under identical conditions, and their crystals thus obtained are isomorphous (Table 3 ▸); it is therefore surprising to find that while the structure of compound (I) is ordered, that of compound (II) is disordered in two different ways, so that although these compounds are isomorphous, they cannot be regarded as strictly isostructural (*cf*. Acosta *et al.*, 2009 ▸; Yépes *et al.*, 2012 ▸). It is also surprising to note that the unit-cell volume, and hence the molar volume, is smaller for the bromo compound (I) than for the chloro compound (II), although the reverse relationship would be expected (Hofmann, 2002 ▸). The larger molar volume for (II) is almost certainly associated with the disorder, but this does not shed any light on the underlying reasons for this disorder, as compared with the ordered structure of (I). Whether the larger volume is a consequence of the disorder or whether the disorder is actually a consequence of the larger molar volume, itself the result of some other factors, remains in doubt. In the absence of a systematic study of the effects of the crystallization regime on relationship between unit-cell volume and the order/disorder question, which we currently have no plans to undertake, any further comments could not be more than pure speculation.

## Supra­molecular features   

The hydrogen bonds formed by compounds (I) and (II) are very similar (Table 2 ▸), so that it is necessary only to discuss in detail the supra­molecular assembly in compound (I). Within the selected asymmetric unit of (I), the two mol­ecules are linked by an N—H⋯O hydrogen bond, and bimolecular units of this type that are related by the *n*-glide plane at *y* = 0.75 are linked by a second, almost linear N—H⋯O hydrogen bond to form a 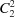
(10) (Etter, 1990 ▸; Etter *et al.*, 1990 ▸; Bernstein *et al.*, 1995 ▸) chain running parallel to the [101] direction. The formation of this chain is augmented by a C—H⋯O hydrogen bond between bimolecular units related by the *n*-glide plane at *y* = 0.75, resulting in a chain of rings running parallel to the [101] direction (Fig. 6 ▸). There is also a C—H⋯π(arene) inter­action within the selected asymmetric unit. Inversion-related pairs of chains of this type are further linked, albeit fairly weakly (Wood *et al.*, 2009 ▸), by a second C—H⋯O hydrogen bond to form a complex sheet lying parallel to (10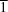). Entirely similar remarks apply to the supra­molecular assembly of compound (II) (Table 2 ▸).

## Database survey   

The structures of several dialkyl propane­diaotes containing pyrazole units in the side-chain at the 2-position have been reported although, in general, these compounds have all been prepared by elaboration of a pre-existing 2-benzyl or 2-benz­yl­idene ester. These structures, whose names are given as those used in the original reports, include those of dimethyl 2-[phen­yl(3-phenyl-1*H*-pyrazol-1-yl)meth­yl]malonate (Jiang *et al.*, 2008 ▸), dimethyl [3,5-dimethyl-1*H*-pyrazol-1-yl(phen­yl)meth­yl]malonate (Meskini, Toupet *et al.*, 2010 ▸), diethyl 2-[phen­yl(pyrazol-1-yl)meth­yl]propane­dioate (Meskini, Daoudi, Daran, Zoulhri *et al.*, 2010 ▸) and diethyl 2-[(3,5-dimethyl-1*H*-pyrzol-1-yl)(4-meth­oxy­phen­yl)meth­yl]propane­dioate (Meskini, Daoudi, Daran, Kerbal *et al.*, 2010 ▸). It is inter­esting that in all of these compounds, the pyrazole unit is linked to the rest of the mol­ecule *via* an N atom, rather than *via* a C atom, as in compounds (I) and (II) reported here. We also note here the recent structure determinations for some 1-aryl-1*H*-pyrazole-3,4-di­carboxyl­ate derivatives (Asma *et al.*, 2018 ▸) and some 4,5-hydro­pyrazole-1-carbo­thio­amides (Shaibah *et al.*, 2020*b* ▸).

## Synthesis and crystallization   

**Figure 5:**
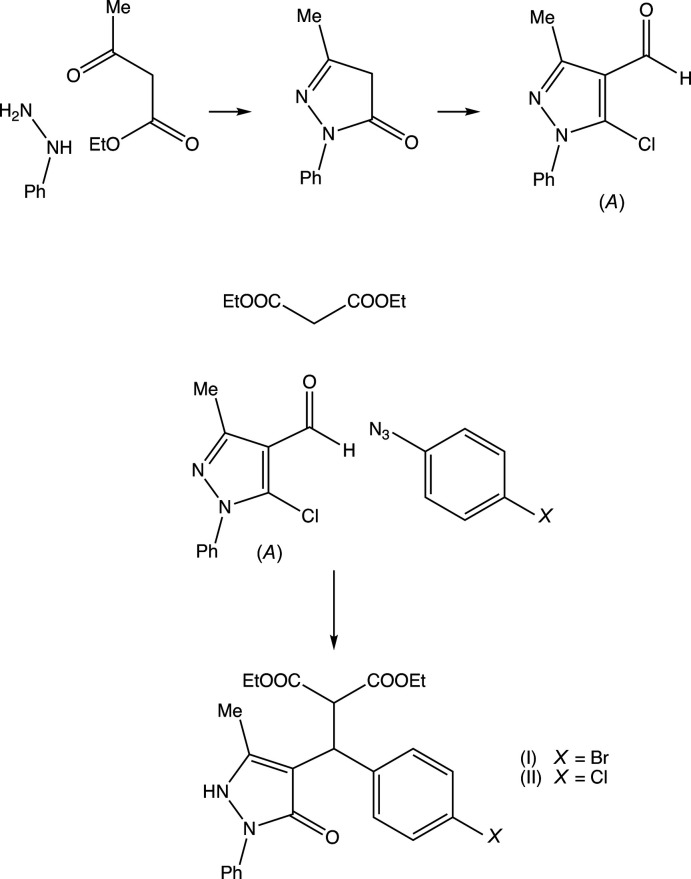
The reaction sequence leading to the formation of compounds (I) and (II).

**Figure 6:**
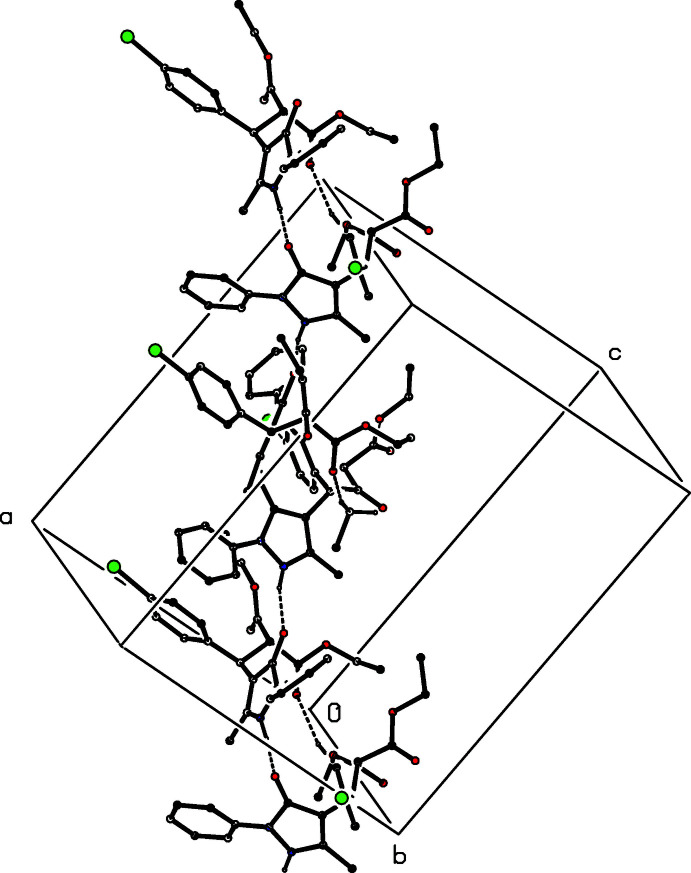
Part of the crystal structure of compound (I), showing the formation of a chain of rings running parallel to the [101] direction and containing N—H⋯O and C—H⋯O hydrogen bonds, all drawn using dashed lines. For the sake of clarity, the H atoms not involved in the motifs shown have been omitted.

The inter­mediate (*A*) (Fig. 5 ▸) was prepared by acid-catalysed cyclo­condensation of phenyl­hydrazine with ethyl 3-oxo­butano­ate (Vogel *et al.*, 2000 ▸), followed by chloro-formyl­ation under Vilsmeier–Haack conditions. For the synthesis of compounds (I) and (II), a mixture of diethyl propandioate (0.15 mmol, 24.0 mg), the pyrazole inter­mediate (*A*, Fig. 5 ▸) (0.10 mmol, 22.3 mg) and either 4-azido­bromo­benzene, for (I) (0.11 mmol, 21.8 mg) or 4-azido­chloro­benzene, for (II) (0.11 mmol, 16.9 g), was heated to 523 K for 5 min in a sealed, evacuated glass tube of volume *ca* 2 ml. After cooling to ambient temperature, the reaction mixtures were added to an excess of cold water, and the resulting solids were collected by filtration, dried in air, and crystallized by slow evaporation, at ambient temperature and in the presence of air, from a solution in *N*,*N*-di­methyl­formamide to give crystals suitable for single-crystal X-ray diffraction.

Compound (I). Yield 40%, m.p. 475–477 K; IR (cm^−1^) 3150 (*br*, NH), 1705 (ring C=O), 1690 (ester C=O); NMR (DMSO-*d*_6_) δ(^1^H) 2.21 (*t*, *J* = 7.2 Hz, 6H, ester CH_3_), 2.30 (*d*, *J* = 5.1 Hz,1H), 2.36 (*s*, 3H, ring CH_3_), 2.54 (*d*, *J* = 5.1 Hz, 1H) 3.98 (*q*, *J* = 7.2 Hz, 4H, CH_2_), 7.1–8.6 (*m*, 9H, aromatic).

Compound (II). Yield 35%, m.p. 444–446 K; IR (cm^−1^) 3230 (br, NH), 1702 (ring C=O), 1605 (ester C=O); NMR (DMSO-*d*_6_) δ(^1^H) 1.78 (*t*, *J* = 7.3 Hz, 6H, ester CH_3_), 2.30 (*s*, 3H, ring CH_3_), 2.45 (*d*, *J* = 5.7 Hz, 1H), 2.83 (*d*, *J* = 5.7 Hz, 1H) 4.02 (*q*, *J* = 7.3 Hz, 4H, CH_2_), 6.8–8.6 (*m*, 9H, aromatic).

## Refinement   

Crystal data, data collection and refinement details are summarized in Table 3 ▸. For compound (I), all H atoms were located in difference maps. The H atoms bonded to C atoms were then treated as riding atoms in geometrically idealized positions with C—H distances of 0.95 Å (aromatic), 0.98 Å (CH_3_), 0.99 Å (CH_2_) or 1.00 Å (aliphatic C—H), and with *U*_iso_(H) = 1.2*U*_eq_(C). For the H atoms bonded to N atoms, the atomic coordinates were refined with *U*_iso_(H) = 1.2*U*_eq_(N) giving the N—H distances shown in Table 2 ▸. A search for possible additional crystallographic symmetry found none. For compound (II), the initial refinement used the atomic coordinates of compound (I), with exactly the same treatment for the H atoms, but it was immediately apparent that both of the independent mol­ecules in (II) exhibited disorder. In mol­ecule 1, containing atom C121, the unsubstituted phenyl ring was disordered, while in mol­ecule 2, containing atom C221, the di­ethyl­malonate fragment was disordered. In each mol­ecule, the bonded distances and the 1,3-non-bonded distances in the minor disorder component were restrained to be the same of the corresponding distances in the major component, subject to s.u. values of 0.01 and 0.02 Å, respectively. In addition, the anisotropic displacement parameters for the atoms in the disordered portions of the mol­ecules were subjected to a similarity restraint, while the C221—C22 and C221—C32 distances were restrained to be equal, subject to an s.u. of 0.02 Å, as were all of the O—C distances and all of the C—C distances in the eth­oxy units. Subject to these conditions, the N—H distances are as shown in Table 2 ▸, and the refined disorder occupancies are 0.635 (10) and 0.365 (10) in mol­ecule 1, and 0.690 (5) and 0.310 (5) in mol­ecule 2.

## Supplementary Material

Crystal structure: contains datablock(s) global, I, II. DOI: 10.1107/S2056989020011676/zl2798sup1.cif

Structure factors: contains datablock(s) I. DOI: 10.1107/S2056989020011676/zl2798Isup2.hkl

Structure factors: contains datablock(s) II. DOI: 10.1107/S2056989020011676/zl2798IIsup3.hkl

Supporting information file. DOI: 10.1107/S2056989020011676/zl2798Isup4.cml

Supporting information file. DOI: 10.1107/S2056989020011676/zl2798IIsup5.cml

CCDC references: 2025336, 2025335

Additional supporting information:  crystallographic information; 3D view; checkCIF report

## Figures and Tables

**Table 1:** Selected torsional angles (°)

Parameter	(I), molecule 1 (*x* = 1)	(I), molecule 2 (*x* = 2)	(II), molecule 1 (*x* = 1)	(II), molecule 2 (*x* = 2)
C*x*1—C*x*2—C*x*21—C*x*31	179.2 (3)	−165.2 (4)	180.0 (2)	−164.7 (6)
C*x*3—C*x*2—C*x*21—C*x*44	170.2 (3)	−170.4 (3)	170.2 (2)	−171.1 (4)
H*x*2—C*x*2—C*x*21—H*x*21	177	−167	178	−168
C31—C32—C221—C231				166.6 (13)
C33—C32—C221—C244				−171.1 (4)
H32—C32—C221—H221				−166
C*x*21—C*x*2—C*x*1—O*x*2	66.6 (5)	−167.1 (4)	67.4 (3)	−169.0 (4)
C*x*2—C*x*1—O*x*2—C*x*4	176.1 (4)	−178.7 (5)	175.9 (3)	178.5 (7)
C*x*1—O*x*2—C*x*4—C*x*5	−83.1 (6)	−95.2 (8)	−83.5 (5)	−87.7 (9)
C221—C22—C31—O32				−150.2 (13)
C22—C31—O32—C34				−170.0 (11)
C31—O32—C34—C35				158.1 (14)
C*x*21—C*x*2—C*x*3—O*x*4	164.5 (3)	102.9 (4)	163.3 (2)	111.7 (13)
C*x*2—C*x*3—O*x*4—C*x*6	177.3 (4)	−176.5 (4)	177.8 (2)	−179.1 (7)
C*x*3—O*x*4—C*x*6—C*x*7	175.9 (5)	154.1 (5)	177.7 (3)	150.7 (13)
C221—C22—C33—O34				95.3 (16)
C22—C33—O34—C36				−174 (2)
C33—O34—C36—C37				96 (2)
C*x*2—C*x*21—C*x*31—C*x*32	155.9 (4)	86.7 (5)	155.0 (2)	85.9 (6)
C*x*2—C*x*21—C*x*44—C*x*45	−113.8 (5)	−135.8 (4)	−117.6 (3)	−133.1 (5)
N*x*41—N*x*42—C*x*51—C*x*52	−159.2 (4)	151.4 (4)	−157.6 (7)	151.4 (3)
N*x*41—N*x*42—C*x*61—C*x*62			−157 (2)	

**Table 2:** Hydrogen-bond parameters (Å, °) *Cg*1 and *Cg*2 represent the centroids of the rings (C151–C156) and (C161–C166).

Compound	*D*—H⋯*A*	*D*—H	H⋯*A*	*D*⋯*A*	*D*—H⋯*A*
(I)	N141—H141⋯O243	0.86 (5)	1.85 (5)	2.678 (5)	162 (4)
	N241—H241⋯O143^i^	0.95 (4)	1.74 (4)	2.690 (5)	175 (4)
	C14—H14⋯O21^ii^	0.99	2.32	3.288 (7)	166
	C132—H132⋯O13^iii^	0.95	2.55	3.359 (5)	144
	C235—H235⋯*Cg*1	0.95	2.64	3.372 (6)	134
					
(II)	N141—H141⋯O243	0.85 (3)	1.89 (3)	2.692 (3)	159 (3)
	N241—H241⋯O143^i^	0.86 (3)	1.85 (3)	2.703 (3)	172 (3)
	C14—H14⋯O21^ii^	0.99	2.38	3.346 (10)	166
	C132—H132⋯O13^iii^	0.95	2.58	3.416 (5)	147
	C235—H235⋯*Cg*1	0.95	2.72	3.439 (9)	133
	C235—H235⋯*Cg*2	0.95	2.72	3.439 (9)	133

Symmetry codes: (i) −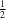 + *x*, 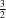 − *y*, −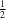 + *z*; (ii) 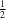 + *x*, 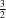 − *y*, 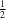 + *z*; (iii) 1 − *x*, 1 − *y*, 1 − *z*.

**Table 3:** Experimental details

	(I)	(II)
Crystal data		
Chemical formula	C_24_H_25_BrN_2_O_5_	C_24_H_25_ClN_2_O_5_
*M*_r_	501.36	456.91
Crystal system, space group	Monoclinic, *P*2_1_/*n*	Monoclinic, *P*2_1_/*n*
Temperature (K)	150	150
*a*, *b*, *c* (Å)	13.5644 (5), 20.3405 (7), 17.4818 (8)	13.5609 (8), 20.280 (1), 17.728 (1)
β (°)	94.858 (4)	95.363 (5)
*V* (Å^3^)	4806.0 (3)	4854.1 (5)
*Z*	8	8
Radiation type	Mo *K*α	Mo *K*α
μ (mm^−1^)	1.75	0.19
Crystal size (mm)	0.44 × 0.32 × 0.24	0.46 × 0.44 × 0.34

Data collection		
Diffractometer	Oxford Diffraction Xcalibur with Sapphire CCD detector	Oxford Diffraction Xcalibur with Sapphire CCD detector
Absorption correction	Multi-scan (*CrysAlis RED*; Oxford Diffraction, 2009 ▸)	Multi-scan (*CrysAlis RED*; Oxford Diffraction, 2009 ▸)
*T*_min_, *T*_max_	0.351, 0.658	0.826, 0.936
No. of measured, independent and observed [*I* > 2σ(*I*)] reflections	20004, 9476, 5653	20936, 9574, 6504
*R*_int_	0.034	0.023
(sin θ/λ)_max_ (Å^−1^)	0.618	0.618

Refinement		
*R*[*F* ^2^ > 2σ(*F* ^2^)], *wR*(*F* ^2^), *S*	0.065, 0.164, 1.03	0.067, 0.192, 1.03
No. of reflections	9476	9574
No. of parameters	589	746
No. of restraints	0	571
H-atom treatment	H atoms treated by a mixture of independent and constrained refinement	H atoms treated by a mixture of independent and constrained refinement
Δρ_max_, Δρ_min_ (e Å^−3^)	1.31, −1.37	1.06, −0.91

Computer programs: *CrysAlis CCD* and *CrysAlis RED* (Oxford Diffraction, 2009 ▸), *SHELXT* (Sheldrick, 2015*a* ▸), *SHELXL2014* (Sheldrick, 2015*b* ▸) and *PLATON* (Spek, 2020 ▸).

## supplementary crystallographic information

### Diethyl (RS)-2-[(4-bromophenyl)(5-methyl-3-oxo-2-phenyl-2,3-dihydro-1H-pyrazol-4-yl)methyl]propanedioate (I) Crystal data

**Table:**

C_24_H_25_BrN_2_O_5_	*F*(000) = 2064
*M**_r_* = 501.36	*D*_x_ = 1.386 Mg m^−^^3^
Monoclinic, *P*2_1_/*n*	Mo *K*α radiation, λ = 0.71073 Å
*a* = 13.5644 (5) Å	Cell parameters from 10428 reflections
*b* = 20.3405 (7) Å	θ = 2.5–27.6°
*c* = 17.4818 (8) Å	µ = 1.75 mm^−^^1^
β = 94.858 (4)°	*T* = 150 K
*V* = 4806.0 (3) Å^3^	Block, orange
*Z* = 8	0.44 × 0.32 × 0.24 mm

### Diethyl (RS)-2-[(4-bromophenyl)(5-methyl-3-oxo-2-phenyl-2,3-dihydro-1H-pyrazol-4-yl)methyl]propanedioate (I) Data collection

**Table:**

Oxford Diffraction Xcalibur with Sapphire CCD detector diffractometer	9476 independent reflections
Radiation source: Enhance (Mo) X-ray Source	5653 reflections with *I* > 2σ(*I*)
Graphite monochromator	*R*_int_ = 0.034
ω scans	θ_max_ = 26.1°, θ_min_ = 2.5°
Absorption correction: multi-scan (CrysAlis RED; Oxford Diffraction, 2009)	*h* = −16→8
*T*_min_ = 0.351, *T*_max_ = 0.658	*k* = −25→25
20004 measured reflections	*l* = −21→21

### Diethyl (RS)-2-[(4-bromophenyl)(5-methyl-3-oxo-2-phenyl-2,3-dihydro-1H-pyrazol-4-yl)methyl]propanedioate (I) Refinement

**Table:**

Refinement on *F*^2^	Primary atom site location: difference Fourier map
Least-squares matrix: full	Hydrogen site location: mixed
*R*[*F*^2^ > 2σ(*F*^2^)] = 0.065	H atoms treated by a mixture of independent and constrained refinement
*wR*(*F*^2^) = 0.164	*w* = 1/[σ^2^(*F*_o_^2^) + (0.0547*P*)^2^ + 8.9767*P*] where *P* = (*F*_o_^2^ + 2*F*_c_^2^)/3
*S* = 1.02	(Δ/σ)_max_ = 0.001
9476 reflections	Δρ_max_ = 1.31 e Å^−^^3^
589 parameters	Δρ_min_ = −1.37 e Å^−^^3^
0 restraints	

### Diethyl (RS)-2-[(4-bromophenyl)(5-methyl-3-oxo-2-phenyl-2,3-dihydro-1H-pyrazol-4-yl)methyl]propanedioate (I) Special details

**Table:**

Geometry. All esds (except the esd in the dihedral angle between two l.s. planes) are estimated using the full covariance matrix. The cell esds are taken into account individually in the estimation of esds in distances, angles and torsion angles; correlations between esds in cell parameters are only used when they are defined by crystal symmetry. An approximate (isotropic) treatment of cell esds is used for estimating esds involving l.s. planes.

### Diethyl (RS)-2-[(4-bromophenyl)(5-methyl-3-oxo-2-phenyl-2,3-dihydro-1H-pyrazol-4-yl)methyl]propanedioate (I) Fractional atomic coordinates and isotropic or equivalent isotropic displacement parameters (Å^2^)

**Table:**

	*x*	*y*	*z*	*U*_iso_*/*U*_eq_	
C11	0.6286 (4)	0.6998 (2)	0.5514 (2)	0.0380 (11)	
C12	0.6748 (3)	0.6325 (2)	0.5677 (2)	0.0308 (10)	
H12	0.7486	0.6358	0.5718	0.037*	
C13	0.6396 (3)	0.6080 (2)	0.6433 (2)	0.0340 (10)	
O11	0.5415 (3)	0.70945 (17)	0.5506 (2)	0.0521 (9)	
O12	0.6944 (3)	0.74418 (17)	0.5355 (2)	0.0536 (9)	
C14	0.6538 (5)	0.8097 (3)	0.5140 (4)	0.0707 (17)	
H14A	0.7059	0.8433	0.5249	0.085*	
H14B	0.5987	0.8199	0.5458	0.085*	
C15	0.6167 (6)	0.8131 (3)	0.4306 (4)	0.086 (2)	
H15A	0.6037	0.8590	0.4160	0.129*	
H15B	0.5555	0.7876	0.4220	0.129*	
H15C	0.6668	0.7948	0.3993	0.129*	
O13	0.5740 (2)	0.56998 (19)	0.64905 (17)	0.0527 (9)	
O14	0.6900 (2)	0.63699 (16)	0.70281 (16)	0.0432 (8)	
C16	0.6583 (4)	0.6189 (3)	0.7788 (3)	0.0529 (14)	
H16A	0.5895	0.6336	0.7833	0.063*	
H16B	0.6611	0.5706	0.7856	0.063*	
C17	0.7260 (6)	0.6513 (4)	0.8377 (3)	0.103 (3)	
H17A	0.7061	0.6404	0.8889	0.154*	
H17B	0.7230	0.6990	0.8303	0.154*	
H17C	0.7937	0.6359	0.8332	0.154*	
C121	0.6388 (3)	0.5868 (2)	0.5004 (2)	0.0281 (9)	
H121	0.5654	0.5832	0.5008	0.034*	
C131	0.6800 (3)	0.5174 (2)	0.5076 (2)	0.0297 (10)	
C132	0.6266 (3)	0.4671 (2)	0.4690 (2)	0.0320 (10)	
H132	0.5664	0.4776	0.4399	0.038*	
C133	0.6580 (3)	0.4031 (2)	0.4718 (3)	0.0393 (11)	
H133	0.6206	0.3697	0.4447	0.047*	
C134	0.7451 (4)	0.3882 (2)	0.5146 (3)	0.0424 (12)	
Br14	0.78948 (5)	0.29969 (3)	0.51980 (4)	0.0718 (2)	
C135	0.7999 (4)	0.4361 (2)	0.5532 (3)	0.0469 (13)	
H135	0.8601	0.4252	0.5822	0.056*	
C136	0.7671 (3)	0.5009 (2)	0.5497 (3)	0.0396 (11)	
H136	0.8051	0.5341	0.5766	0.047*	
N141	0.6353 (3)	0.66770 (19)	0.3126 (2)	0.0343 (9)	
H141	0.610 (3)	0.682 (2)	0.269 (3)	0.041*	
N142	0.7359 (2)	0.66438 (17)	0.33052 (19)	0.0308 (8)	
C143	0.7531 (3)	0.6347 (2)	0.4028 (2)	0.0287 (9)	
O143	0.8375 (2)	0.62747 (14)	0.43643 (16)	0.0325 (7)	
C144	0.6588 (3)	0.6177 (2)	0.4248 (2)	0.0264 (9)	
C145	0.5892 (3)	0.6390 (2)	0.3682 (2)	0.0322 (10)	
C146	0.4794 (3)	0.6345 (3)	0.3619 (3)	0.0482 (13)	
H16C	0.4540	0.6531	0.4081	0.072*	
H16D	0.4594	0.5883	0.3566	0.072*	
H16E	0.4523	0.6591	0.3167	0.072*	
C151	0.8024 (3)	0.7010 (2)	0.2889 (2)	0.0352 (11)	
C152	0.8992 (4)	0.6819 (3)	0.2904 (3)	0.0458 (12)	
H152	0.9223	0.6439	0.3181	0.055*	
C153	0.9637 (4)	0.7202 (3)	0.2496 (3)	0.0624 (16)	
H153	1.0316	0.7086	0.2504	0.075*	
C154	0.9283 (5)	0.7744 (3)	0.2086 (3)	0.0620 (17)	
H154	0.9723	0.8001	0.1815	0.074*	
C155	0.8322 (5)	0.7912 (3)	0.2064 (3)	0.0530 (14)	
H155	0.8084	0.8277	0.1764	0.064*	
C156	0.7683 (4)	0.7560 (2)	0.2476 (2)	0.0416 (12)	
H156	0.7012	0.7691	0.2477	0.050*	
C21	0.3724 (4)	0.5780 (2)	0.1386 (3)	0.0461 (13)	
C22	0.4833 (3)	0.5826 (2)	0.1456 (3)	0.0343 (10)	
H22	0.5039	0.6119	0.1901	0.041*	
C23	0.5243 (3)	0.5147 (2)	0.1631 (3)	0.0361 (11)	
O21	0.3189 (2)	0.58178 (17)	0.0809 (2)	0.0564 (10)	
O22	0.3409 (3)	0.5652 (2)	0.2086 (2)	0.0703 (11)	
C24	0.2348 (5)	0.5572 (4)	0.2151 (5)	0.099 (2)	
H24A	0.2024	0.5424	0.1652	0.118*	
H24B	0.2243	0.5229	0.2538	0.118*	
C25	0.1915 (6)	0.6164 (5)	0.2368 (5)	0.122 (3)	
H25A	0.1202	0.6100	0.2392	0.183*	
H25B	0.2026	0.6506	0.1989	0.183*	
H25C	0.2214	0.6300	0.2873	0.183*	
O23	0.4973 (2)	0.46590 (15)	0.12862 (19)	0.0445 (8)	
O24	0.5950 (2)	0.51763 (15)	0.21995 (18)	0.0452 (8)	
C26	0.6462 (4)	0.4563 (3)	0.2406 (3)	0.0580 (15)	
H26A	0.6093	0.4312	0.2775	0.070*	
H26B	0.6514	0.4288	0.1944	0.070*	
C27	0.7443 (4)	0.4732 (3)	0.2752 (4)	0.085 (2)	
H27A	0.7795	0.4330	0.2923	0.128*	
H27B	0.7382	0.5022	0.3193	0.128*	
H27C	0.7814	0.4958	0.2372	0.128*	
C221	0.5227 (3)	0.6125 (2)	0.0726 (2)	0.0342 (10)	
H221	0.4887	0.5892	0.0274	0.041*	
C231	0.6330 (3)	0.6012 (2)	0.0704 (2)	0.0318 (10)	
C232	0.6673 (4)	0.5438 (2)	0.0396 (3)	0.0423 (12)	
H232	0.6211	0.5132	0.0162	0.051*	
C233	0.7673 (4)	0.5297 (3)	0.0421 (3)	0.0509 (13)	
H233	0.7894	0.4898	0.0210	0.061*	
C234	0.8341 (4)	0.5739 (3)	0.0755 (3)	0.0454 (12)	
Br24	0.97147 (4)	0.55439 (3)	0.08460 (4)	0.0741 (2)	
C235	0.8021 (4)	0.6334 (3)	0.1025 (3)	0.0484 (13)	
H235	0.8485	0.6653	0.1226	0.058*	
C236	0.7021 (3)	0.6460 (2)	0.0999 (3)	0.0402 (11)	
H236	0.6803	0.6867	0.1190	0.048*	
N241	0.4437 (3)	0.77933 (18)	0.0153 (2)	0.0345 (9)	
H241	0.403 (3)	0.811 (2)	−0.012 (3)	0.041*	
N242	0.4662 (3)	0.78984 (17)	0.09295 (19)	0.0333 (9)	
C243	0.5007 (3)	0.7315 (2)	0.1261 (3)	0.0357 (11)	
O243	0.5320 (3)	0.72836 (15)	0.19576 (17)	0.0469 (9)	
C244	0.4930 (3)	0.6839 (2)	0.0664 (2)	0.0350 (10)	
C245	0.4556 (3)	0.7151 (2)	0.0011 (2)	0.0322 (10)	
C246	0.4277 (4)	0.6870 (2)	−0.0763 (3)	0.0503 (13)	
H26C	0.3989	0.6432	−0.0707	0.075*	
H26D	0.4867	0.6835	−0.1047	0.075*	
H26E	0.3791	0.7156	−0.1043	0.075*	
C251	0.4786 (3)	0.8545 (2)	0.1224 (3)	0.0335 (10)	
C252	0.4571 (4)	0.8667 (2)	0.1973 (3)	0.0463 (12)	
H252	0.4323	0.8326	0.2275	0.056*	
C253	0.4722 (4)	0.9291 (3)	0.2275 (3)	0.0581 (15)	
H253	0.4585	0.9378	0.2789	0.070*	
C254	0.5068 (4)	0.9786 (3)	0.1836 (3)	0.0551 (15)	
H254	0.5167	1.0213	0.2048	0.066*	
C255	0.5271 (4)	0.9666 (2)	0.1094 (3)	0.0507 (14)	
H255	0.5507	1.0011	0.0792	0.061*	
C256	0.5132 (3)	0.9043 (2)	0.0783 (3)	0.0403 (11)	
H256	0.5275	0.8959	0.0269	0.048*	

### Diethyl (RS)-2-[(4-bromophenyl)(5-methyl-3-oxo-2-phenyl-2,3-dihydro-1H-pyrazol-4-yl)methyl]propanedioate (I) Atomic displacement parameters (Å^2^)

**Table:**

	*U*^11^	*U*^22^	*U*^33^	*U*^12^	*U*^13^	*U*^23^
C11	0.050 (3)	0.038 (3)	0.027 (2)	−0.007 (2)	0.008 (2)	−0.007 (2)
C12	0.032 (2)	0.035 (3)	0.026 (2)	−0.0067 (19)	0.0016 (17)	−0.0032 (19)
C13	0.036 (2)	0.040 (3)	0.025 (2)	0.001 (2)	0.0002 (19)	−0.002 (2)
O11	0.051 (2)	0.048 (2)	0.059 (2)	0.0100 (17)	0.0144 (17)	0.0055 (18)
O12	0.059 (2)	0.036 (2)	0.066 (2)	−0.0092 (17)	0.0056 (18)	−0.0056 (18)
C14	0.088 (5)	0.041 (3)	0.082 (5)	−0.006 (3)	−0.001 (4)	−0.004 (3)
C15	0.127 (6)	0.059 (4)	0.071 (4)	0.014 (4)	−0.002 (4)	0.005 (4)
O13	0.051 (2)	0.076 (3)	0.0313 (18)	−0.030 (2)	0.0052 (15)	0.0012 (18)
O14	0.053 (2)	0.053 (2)	0.0233 (16)	−0.0141 (16)	0.0003 (14)	−0.0049 (15)
C16	0.068 (4)	0.061 (4)	0.030 (3)	−0.005 (3)	0.007 (2)	0.003 (3)
C17	0.132 (7)	0.140 (7)	0.035 (3)	−0.047 (6)	−0.001 (4)	−0.010 (4)
C121	0.027 (2)	0.035 (2)	0.023 (2)	−0.0067 (18)	0.0005 (16)	0.0011 (19)
C131	0.032 (2)	0.032 (2)	0.025 (2)	−0.0071 (19)	0.0035 (17)	0.0034 (19)
C132	0.031 (2)	0.036 (3)	0.029 (2)	−0.007 (2)	0.0042 (18)	−0.005 (2)
C133	0.044 (3)	0.038 (3)	0.037 (3)	−0.012 (2)	0.012 (2)	−0.011 (2)
C134	0.051 (3)	0.029 (3)	0.049 (3)	−0.003 (2)	0.010 (2)	0.001 (2)
Br14	0.0812 (5)	0.0333 (3)	0.1013 (5)	0.0074 (3)	0.0107 (4)	0.0024 (3)
C135	0.042 (3)	0.040 (3)	0.056 (3)	0.003 (2)	−0.011 (2)	0.003 (3)
C136	0.040 (3)	0.036 (3)	0.040 (3)	−0.004 (2)	−0.009 (2)	−0.002 (2)
N141	0.034 (2)	0.041 (2)	0.0264 (19)	−0.0046 (18)	−0.0051 (16)	0.0080 (18)
N142	0.036 (2)	0.030 (2)	0.0264 (18)	−0.0049 (16)	0.0019 (15)	0.0049 (16)
C143	0.035 (2)	0.021 (2)	0.029 (2)	−0.0025 (19)	−0.0009 (18)	−0.0066 (18)
O143	0.0299 (16)	0.0314 (17)	0.0348 (16)	−0.0037 (13)	−0.0050 (13)	−0.0006 (14)
C144	0.031 (2)	0.025 (2)	0.022 (2)	−0.0061 (18)	−0.0023 (17)	−0.0005 (18)
C145	0.031 (2)	0.036 (3)	0.029 (2)	−0.004 (2)	−0.0021 (18)	−0.001 (2)
C146	0.035 (3)	0.067 (4)	0.041 (3)	−0.008 (2)	−0.007 (2)	0.009 (3)
C151	0.046 (3)	0.034 (3)	0.026 (2)	−0.017 (2)	0.0057 (19)	−0.005 (2)
C152	0.045 (3)	0.046 (3)	0.047 (3)	−0.015 (2)	0.011 (2)	−0.009 (2)
C153	0.057 (3)	0.068 (4)	0.066 (4)	−0.018 (3)	0.028 (3)	−0.030 (3)
C154	0.090 (5)	0.056 (4)	0.044 (3)	−0.040 (4)	0.025 (3)	−0.011 (3)
C155	0.079 (4)	0.046 (3)	0.035 (3)	−0.027 (3)	0.011 (3)	−0.006 (2)
C156	0.065 (3)	0.033 (3)	0.027 (2)	−0.019 (2)	0.005 (2)	0.000 (2)
C21	0.039 (3)	0.030 (3)	0.067 (4)	0.006 (2)	−0.007 (3)	−0.011 (3)
C22	0.039 (3)	0.024 (2)	0.039 (3)	0.0002 (19)	−0.002 (2)	−0.001 (2)
C23	0.036 (3)	0.030 (3)	0.042 (3)	−0.003 (2)	0.002 (2)	0.008 (2)
O21	0.045 (2)	0.042 (2)	0.078 (3)	0.0055 (17)	−0.0207 (19)	−0.0125 (19)
O22	0.041 (2)	0.094 (3)	0.076 (3)	−0.003 (2)	0.0119 (19)	−0.016 (3)
C24	0.045 (4)	0.104 (6)	0.149 (7)	0.001 (4)	0.016 (4)	−0.021 (5)
C25	0.070 (5)	0.140 (8)	0.156 (8)	0.022 (5)	0.011 (5)	−0.026 (7)
O23	0.049 (2)	0.0285 (18)	0.054 (2)	−0.0010 (15)	−0.0072 (16)	−0.0006 (16)
O24	0.0442 (19)	0.0356 (19)	0.053 (2)	0.0014 (15)	−0.0131 (16)	0.0060 (16)
C26	0.051 (3)	0.047 (3)	0.073 (4)	0.009 (3)	−0.009 (3)	0.014 (3)
C27	0.061 (4)	0.084 (5)	0.107 (5)	0.005 (4)	−0.018 (4)	0.029 (4)
C221	0.046 (3)	0.025 (2)	0.029 (2)	0.002 (2)	−0.0070 (19)	0.000 (2)
C231	0.047 (3)	0.025 (2)	0.022 (2)	−0.001 (2)	−0.0022 (19)	0.0038 (19)
C232	0.047 (3)	0.035 (3)	0.043 (3)	−0.002 (2)	−0.004 (2)	−0.009 (2)
C233	0.053 (3)	0.043 (3)	0.058 (3)	0.000 (3)	0.009 (3)	−0.012 (3)
C234	0.042 (3)	0.048 (3)	0.046 (3)	0.001 (2)	0.004 (2)	−0.006 (3)
Br24	0.0468 (3)	0.0812 (5)	0.0953 (5)	0.0012 (3)	0.0118 (3)	−0.0229 (4)
C235	0.052 (3)	0.046 (3)	0.047 (3)	−0.013 (3)	0.003 (2)	−0.019 (3)
C236	0.046 (3)	0.034 (3)	0.040 (3)	0.000 (2)	0.001 (2)	−0.012 (2)
N241	0.042 (2)	0.029 (2)	0.031 (2)	0.0054 (17)	−0.0103 (16)	0.0030 (17)
N242	0.042 (2)	0.026 (2)	0.0300 (19)	0.0036 (17)	−0.0079 (16)	0.0003 (16)
C243	0.046 (3)	0.023 (2)	0.036 (3)	0.004 (2)	−0.008 (2)	0.005 (2)
O243	0.077 (2)	0.0295 (18)	0.0304 (17)	0.0088 (17)	−0.0155 (16)	0.0010 (14)
C244	0.038 (3)	0.027 (2)	0.038 (3)	0.001 (2)	−0.007 (2)	0.001 (2)
C245	0.035 (2)	0.032 (3)	0.029 (2)	0.002 (2)	−0.0023 (18)	0.001 (2)
C246	0.070 (4)	0.043 (3)	0.036 (3)	0.006 (3)	−0.013 (2)	−0.005 (2)
C251	0.034 (2)	0.025 (2)	0.040 (3)	0.0055 (19)	−0.0082 (19)	−0.002 (2)
C252	0.059 (3)	0.034 (3)	0.046 (3)	0.003 (2)	0.003 (2)	−0.003 (2)
C253	0.069 (4)	0.044 (3)	0.060 (4)	0.008 (3)	−0.003 (3)	−0.013 (3)
C254	0.057 (3)	0.031 (3)	0.074 (4)	0.002 (3)	−0.015 (3)	−0.010 (3)
C255	0.051 (3)	0.032 (3)	0.065 (4)	−0.003 (2)	−0.016 (3)	0.009 (3)
C256	0.044 (3)	0.030 (3)	0.045 (3)	−0.001 (2)	−0.009 (2)	0.004 (2)

### Diethyl (RS)-2-[(4-bromophenyl)(5-methyl-3-oxo-2-phenyl-2,3-dihydro-1H-pyrazol-4-yl)methyl]propanedioate (I) Geometric parameters (Å, º)

**Table:**

C11—O11	1.196 (5)	C21—O21	1.194 (6)
C11—O12	1.315 (5)	C21—O22	1.356 (6)
C11—C12	1.524 (6)	C21—C22	1.501 (6)
C12—C13	1.526 (6)	C22—C23	1.511 (6)
C12—C121	1.545 (5)	C22—C221	1.549 (6)
C12—H12	1.0000	C22—H22	1.0000
C13—O13	1.189 (5)	C23—O23	1.202 (5)
C13—O14	1.333 (5)	C23—O24	1.323 (5)
O12—C14	1.478 (6)	O22—C24	1.462 (7)
C14—C15	1.504 (8)	C24—C25	1.407 (10)
C14—H14A	0.9900	C24—H24A	0.9900
C14—H14B	0.9900	C24—H24B	0.9900
C15—H15A	0.9800	C25—H25A	0.9800
C15—H15B	0.9800	C25—H25B	0.9800
C15—H15C	0.9800	C25—H25C	0.9800
O14—C16	1.477 (5)	O24—C26	1.459 (6)
C16—C17	1.475 (7)	C26—C27	1.456 (8)
C16—H16A	0.9900	C26—H26A	0.9900
C16—H16B	0.9900	C26—H26B	0.9900
C17—H17A	0.9800	C27—H27A	0.9800
C17—H17B	0.9800	C27—H27B	0.9800
C17—H17C	0.9800	C27—H27C	0.9800
C121—C144	1.508 (5)	C221—C244	1.510 (6)
C121—C131	1.521 (6)	C221—C231	1.518 (6)
C121—H121	1.0000	C221—H221	1.0000
C131—C136	1.379 (6)	C231—C236	1.376 (6)
C131—C132	1.393 (6)	C231—C232	1.384 (6)
C132—C133	1.369 (6)	C232—C233	1.382 (7)
C132—H132	0.9500	C232—H232	0.9500
C133—C134	1.379 (7)	C233—C234	1.371 (7)
C133—H133	0.9500	C233—H233	0.9500
C134—C135	1.369 (7)	C234—C235	1.384 (7)
C134—Br14	1.897 (5)	C234—Br24	1.898 (5)
C135—C136	1.389 (6)	C235—C236	1.377 (7)
C135—H135	0.9500	C235—H235	0.9500
C136—H136	0.9500	C236—H236	0.9500
N141—C145	1.334 (5)	N241—C245	1.342 (5)
N141—N142	1.376 (5)	N241—N242	1.382 (5)
N141—H141	0.86 (5)	N241—H241	0.95 (5)
N142—C143	1.402 (5)	N242—C243	1.385 (5)
N142—C151	1.417 (5)	N242—C251	1.418 (5)
C143—O143	1.250 (5)	C243—O243	1.257 (5)
C143—C144	1.409 (6)	C243—C244	1.420 (6)
C144—C145	1.378 (5)	C244—C245	1.367 (6)
C145—C146	1.488 (6)	C245—C246	1.487 (6)
C146—H16C	0.9800	C246—H26C	0.9800
C146—H16D	0.9800	C246—H26D	0.9800
C146—H16E	0.9800	C246—H26E	0.9800
C151—C152	1.368 (7)	C251—C256	1.378 (6)
C151—C156	1.389 (6)	C251—C252	1.388 (6)
C152—C153	1.409 (7)	C252—C253	1.383 (7)
C152—H152	0.9500	C252—H252	0.9500
C153—C154	1.380 (8)	C253—C254	1.371 (8)
C153—H153	0.9500	C253—H253	0.9500
C154—C155	1.345 (8)	C254—C255	1.371 (8)
C154—H154	0.9500	C254—H254	0.9500
C155—C156	1.374 (7)	C255—C256	1.385 (7)
C155—H155	0.9500	C255—H255	0.9500
C156—H156	0.9500	C256—H256	0.9500
			
O11—C11—O12	124.9 (5)	O21—C21—O22	124.1 (5)
O11—C11—C12	122.7 (4)	O21—C21—C22	126.7 (5)
O12—C11—C12	112.4 (4)	O22—C21—C22	109.1 (4)
C11—C12—C13	107.6 (4)	C21—C22—C23	108.0 (4)
C11—C12—C121	107.4 (3)	C21—C22—C221	111.7 (4)
C13—C12—C121	111.2 (3)	C23—C22—C221	112.2 (4)
C11—C12—H12	110.2	C21—C22—H22	108.3
C13—C12—H12	110.2	C23—C22—H22	108.3
C121—C12—H12	110.2	C221—C22—H22	108.3
O13—C13—O14	124.0 (4)	O23—C23—O24	125.7 (4)
O13—C13—C12	125.2 (4)	O23—C23—C22	124.3 (4)
O14—C13—C12	110.7 (4)	O24—C23—C22	110.0 (4)
C11—O12—C14	115.3 (4)	C21—O22—C24	118.6 (5)
O12—C14—C15	112.0 (5)	C25—C24—O22	111.2 (6)
O12—C14—H14A	109.2	C25—C24—H24A	109.4
C15—C14—H14A	109.2	O22—C24—H24A	109.4
O12—C14—H14B	109.2	C25—C24—H24B	109.4
C15—C14—H14B	109.2	O22—C24—H24B	109.4
H14A—C14—H14B	107.9	H24A—C24—H24B	108.0
C14—C15—H15A	109.5	C24—C25—H25A	109.5
C14—C15—H15B	109.5	C24—C25—H25B	109.5
H15A—C15—H15B	109.5	H25A—C25—H25B	109.5
C14—C15—H15C	109.5	C24—C25—H25C	109.5
H15A—C15—H15C	109.5	H25A—C25—H25C	109.5
H15B—C15—H15C	109.5	H25B—C25—H25C	109.5
C13—O14—C16	115.0 (4)	C23—O24—C26	116.6 (4)
C17—C16—O14	107.8 (4)	C27—C26—O24	107.4 (5)
C17—C16—H16A	110.1	C27—C26—H26A	110.2
O14—C16—H16A	110.1	O24—C26—H26A	110.2
C17—C16—H16B	110.1	C27—C26—H26B	110.2
O14—C16—H16B	110.1	O24—C26—H26B	110.2
H16A—C16—H16B	108.5	H26A—C26—H26B	108.5
C16—C17—H17A	109.5	C26—C27—H27A	109.5
C16—C17—H17B	109.5	C26—C27—H27B	109.5
H17A—C17—H17B	109.5	H27A—C27—H27B	109.5
C16—C17—H17C	109.5	C26—C27—H27C	109.5
H17A—C17—H17C	109.5	H27A—C27—H27C	109.5
H17B—C17—H17C	109.5	H27B—C27—H27C	109.5
C144—C121—C131	111.6 (3)	C244—C221—C231	113.6 (4)
C144—C121—C12	110.4 (3)	C244—C221—C22	109.2 (4)
C131—C121—C12	113.9 (3)	C231—C221—C22	111.8 (3)
C144—C121—H121	106.9	C244—C221—H221	107.3
C131—C121—H121	106.9	C231—C221—H221	107.3
C12—C121—H121	106.9	C22—C221—H221	107.3
C136—C131—C132	117.8 (4)	C236—C231—C232	117.6 (4)
C136—C131—C121	124.2 (4)	C236—C231—C221	122.2 (4)
C132—C131—C121	118.0 (4)	C232—C231—C221	120.2 (4)
C133—C132—C131	122.2 (4)	C233—C232—C231	121.6 (4)
C133—C132—H132	118.9	C233—C232—H232	119.2
C131—C132—H132	118.9	C231—C232—H232	119.2
C132—C133—C134	118.6 (4)	C234—C233—C232	119.3 (5)
C132—C133—H133	120.7	C234—C233—H233	120.4
C134—C133—H133	120.7	C232—C233—H233	120.4
C135—C134—C133	121.0 (4)	C233—C234—C235	120.2 (5)
C135—C134—Br14	119.7 (4)	C233—C234—Br24	120.5 (4)
C133—C134—Br14	119.3 (4)	C235—C234—Br24	119.2 (4)
C134—C135—C136	119.7 (4)	C236—C235—C234	119.2 (4)
C134—C135—H135	120.2	C236—C235—H235	120.4
C136—C135—H135	120.2	C234—C235—H235	120.4
C131—C136—C135	120.7 (4)	C231—C236—C235	121.8 (4)
C131—C136—H136	119.6	C231—C236—H236	119.1
C135—C136—H136	119.6	C235—C236—H236	119.1
C145—N141—N142	109.3 (3)	C245—N241—N242	108.3 (3)
C145—N141—H141	128 (3)	C245—N241—H241	130 (3)
N142—N141—H141	122 (3)	N242—N241—H241	117 (3)
N141—N142—C143	108.2 (3)	N241—N242—C243	108.5 (3)
N141—N142—C151	121.3 (3)	N241—N242—C251	120.6 (3)
C143—N142—C151	128.7 (3)	C243—N242—C251	128.2 (3)
O143—C143—N142	123.4 (4)	O243—C243—N242	121.6 (4)
O143—C143—C144	131.2 (4)	O243—C243—C244	132.5 (4)
N142—C143—C144	105.4 (3)	N242—C243—C244	106.0 (4)
C145—C144—C143	108.0 (4)	C245—C244—C243	107.3 (4)
C145—C144—C121	126.6 (4)	C245—C244—C221	125.8 (4)
C143—C144—C121	125.2 (3)	C243—C244—C221	126.9 (4)
N141—C145—C144	109.0 (4)	N241—C245—C244	109.8 (4)
N141—C145—C146	120.0 (4)	N241—C245—C246	121.3 (4)
C144—C145—C146	131.0 (4)	C244—C245—C246	129.0 (4)
C145—C146—H16C	109.5	C245—C246—H26C	109.5
C145—C146—H16D	109.5	C245—C246—H26D	109.5
H16C—C146—H16D	109.5	H26C—C246—H26D	109.5
C145—C146—H16E	109.5	C245—C246—H26E	109.5
H16C—C146—H16E	109.5	H26C—C246—H26E	109.5
H16D—C146—H16E	109.5	H26D—C246—H26E	109.5
C152—C151—C156	121.1 (4)	C256—C251—C252	120.4 (4)
C152—C151—N142	119.5 (4)	C256—C251—N242	120.9 (4)
C156—C151—N142	119.5 (4)	C252—C251—N242	118.7 (4)
C151—C152—C153	118.0 (5)	C253—C252—C251	119.2 (5)
C151—C152—H152	121.0	C253—C252—H252	120.4
C153—C152—H152	121.0	C251—C252—H252	120.4
C154—C153—C152	120.1 (6)	C254—C253—C252	120.4 (5)
C154—C153—H153	120.0	C254—C253—H253	119.8
C152—C153—H153	120.0	C252—C253—H253	119.8
C155—C154—C153	120.8 (5)	C253—C254—C255	120.3 (5)
C155—C154—H154	119.6	C253—C254—H254	119.9
C153—C154—H154	119.6	C255—C254—H254	119.9
C154—C155—C156	120.3 (5)	C254—C255—C256	120.1 (5)
C154—C155—H155	119.8	C254—C255—H255	119.9
C156—C155—H155	119.8	C256—C255—H255	119.9
C155—C156—C151	119.7 (5)	C251—C256—C255	119.6 (5)
C155—C156—H156	120.1	C251—C256—H256	120.2
C151—C156—H156	120.1	C255—C256—H256	120.2
			
O11—C11—C12—C13	−53.2 (5)	O21—C21—C22—C23	−107.6 (5)
O12—C11—C12—C13	129.4 (4)	O22—C21—C22—C23	69.1 (5)
O11—C11—C12—C121	66.6 (5)	O21—C21—C22—C221	16.2 (7)
O12—C11—C12—C121	−110.9 (4)	O22—C21—C22—C221	−167.1 (4)
C11—C12—C13—O13	99.4 (5)	C21—C22—C23—O23	47.9 (6)
C121—C12—C13—O13	−17.9 (6)	C221—C22—C23—O23	−75.6 (6)
C11—C12—C13—O14	−78.2 (4)	C21—C22—C23—O24	−133.6 (4)
C121—C12—C13—O14	164.5 (3)	C221—C22—C23—O24	102.9 (4)
O11—C11—O12—C14	−1.2 (7)	O21—C21—O22—C24	−1.9 (8)
C12—C11—O12—C14	176.1 (4)	C22—C21—O22—C24	−178.7 (5)
C11—O12—C14—C15	−83.1 (6)	C21—O22—C24—C25	−95.2 (8)
O13—C13—O14—C16	−0.4 (7)	O23—C23—O24—C26	1.9 (7)
C12—C13—O14—C16	177.3 (4)	C22—C23—O24—C26	−176.5 (4)
C13—O14—C16—C17	175.9 (5)	C23—O24—C26—C27	154.1 (5)
C11—C12—C121—C144	52.8 (4)	C21—C22—C221—C244	68.2 (5)
C13—C12—C121—C144	170.2 (3)	C23—C22—C221—C244	−170.4 (3)
C11—C12—C121—C131	179.2 (3)	C21—C22—C221—C231	−165.2 (4)
C13—C12—C121—C131	−63.4 (4)	C23—C22—C221—C231	−43.8 (5)
C144—C121—C131—C136	101.4 (5)	C244—C221—C231—C236	32.4 (6)
C12—C121—C131—C136	−24.4 (6)	C22—C221—C231—C236	−91.8 (5)
C144—C121—C131—C132	−78.3 (5)	C244—C221—C231—C232	−149.2 (4)
C12—C121—C131—C132	155.9 (4)	C22—C221—C231—C232	86.7 (5)
C136—C131—C132—C133	−0.1 (6)	C236—C231—C232—C233	3.6 (7)
C121—C131—C132—C133	179.6 (4)	C221—C231—C232—C233	−174.9 (4)
C131—C132—C133—C134	0.4 (7)	C231—C232—C233—C234	−0.4 (8)
C132—C133—C134—C135	−0.6 (7)	C232—C233—C234—C235	−3.5 (8)
C132—C133—C134—Br14	179.5 (3)	C232—C233—C234—Br24	176.8 (4)
C133—C134—C135—C136	0.5 (8)	C233—C234—C235—C236	4.1 (8)
Br14—C134—C135—C136	−179.7 (4)	Br24—C234—C235—C236	−176.3 (4)
C132—C131—C136—C135	0.0 (7)	C232—C231—C236—C235	−3.1 (7)
C121—C131—C136—C135	−179.8 (4)	C221—C231—C236—C235	175.4 (4)
C134—C135—C136—C131	−0.1 (8)	C234—C235—C236—C231	−0.7 (7)
C145—N141—N142—C143	−2.8 (5)	C245—N241—N242—C243	5.1 (5)
C145—N141—N142—C151	−168.6 (4)	C245—N241—N242—C251	167.8 (4)
N141—N142—C143—O143	−176.5 (4)	N241—N242—C243—O243	175.8 (4)
C151—N142—C143—O143	−12.1 (7)	C251—N242—C243—O243	14.7 (7)
N141—N142—C143—C144	2.8 (4)	N241—N242—C243—C244	−3.3 (5)
C151—N142—C143—C144	167.3 (4)	C251—N242—C243—C244	−164.4 (4)
O143—C143—C144—C145	177.4 (4)	O243—C243—C244—C245	−178.5 (5)
N142—C143—C144—C145	−1.9 (5)	N242—C243—C244—C245	0.4 (5)
O143—C143—C144—C121	1.9 (7)	O243—C243—C244—C221	0.1 (9)
N142—C143—C144—C121	−177.3 (4)	N242—C243—C244—C221	179.1 (4)
C131—C121—C144—C145	118.5 (5)	C231—C221—C244—C245	98.6 (5)
C12—C121—C144—C145	−113.8 (5)	C22—C221—C244—C245	−135.8 (4)
C131—C121—C144—C143	−66.9 (5)	C231—C221—C244—C243	−79.8 (6)
C12—C121—C144—C143	60.8 (5)	C22—C221—C244—C243	45.8 (6)
N142—N141—C145—C144	1.6 (5)	N242—N241—C245—C244	−4.8 (5)
N142—N141—C145—C146	−178.5 (4)	N242—N241—C245—C246	174.0 (4)
C143—C144—C145—N141	0.2 (5)	C243—C244—C245—N241	2.7 (5)
C121—C144—C145—N141	175.6 (4)	C221—C244—C245—N241	−175.9 (4)
C143—C144—C145—C146	−179.7 (5)	C243—C244—C245—C246	−176.0 (5)
C121—C144—C145—C146	−4.3 (8)	C221—C244—C245—C246	5.4 (8)
N141—N142—C151—C152	−159.2 (4)	N241—N242—C251—C256	−29.9 (6)
C143—N142—C151—C152	38.2 (6)	C243—N242—C251—C256	129.2 (5)
N141—N142—C151—C156	21.3 (6)	N241—N242—C251—C252	151.4 (4)
C143—N142—C151—C156	−141.4 (4)	C243—N242—C251—C252	−49.5 (6)
C156—C151—C152—C153	0.8 (7)	C256—C251—C252—C253	−0.9 (7)
N142—C151—C152—C153	−178.7 (4)	N242—C251—C252—C253	177.8 (4)
C151—C152—C153—C154	−1.2 (7)	C251—C252—C253—C254	0.8 (8)
C152—C153—C154—C155	−0.4 (8)	C252—C253—C254—C255	−0.2 (8)
C153—C154—C155—C156	2.4 (8)	C253—C254—C255—C256	−0.4 (8)
C154—C155—C156—C151	−2.7 (7)	C252—C251—C256—C255	0.4 (7)
C152—C151—C156—C155	1.1 (7)	N242—C251—C256—C255	−178.3 (4)
N142—C151—C156—C155	−179.3 (4)	C254—C255—C256—C251	0.3 (7)

### Diethyl (RS)-2-[(4-bromophenyl)(5-methyl-3-oxo-2-phenyl-2,3-dihydro-1H-pyrazol-4-yl)methyl]propanedioate (I) Hydrogen-bond geometry (Å, º)

**Table:**

*D*—H···*A*	*D*—H	H···*A*	*D*···*A*	*D*—H···*A*
N141—H141···O243	0.86 (5)	1.85 (5)	2.678 (5)	162 (4)
N241—H241···O143^i^	0.95 (4)	1.74 (4)	2.690 (5)	175 (4)
C14—H14*A*···O21^ii^	0.99	2.32	3.288 (7)	166
C132—H132···O13^iii^	0.95	2.55	3.359 (5)	144
C235—H235···*Cg*1	0.95	2.64	3.372 (6)	134

Symmetry codes: (i) *x*−1/2, −*y*+3/2, *z*−1/2; (ii) *x*+1/2, −*y*+3/2, *z*+1/2; (iii) −*x*+1, −*y*+1, −*z*+1.

### Diethyl (RS)-2-[(4-chlorophenyl)(5-methyl-3-oxo-2-phenyl-2,3-dihydro-1H-pyrazol-4-yl)methyl]propanedioate (II) Crystal data

**Table:**

C_24_H_25_ClN_2_O_5_	*F*(000) = 1920
*M**_r_* = 456.91	*D*_x_ = 1.250 Mg m^−^^3^
Monoclinic, *P*2_1_/*n*	Mo *K*α radiation, λ = 0.71073 Å
*a* = 13.5609 (8) Å	Cell parameters from 10261 reflections
*b* = 20.280 (1) Å	θ = 2.5–27.8°
*c* = 17.728 (1) Å	µ = 0.19 mm^−^^1^
β = 95.363 (5)°	*T* = 150 K
*V* = 4854.1 (5) Å^3^	Block, orange
*Z* = 8	0.46 × 0.44 × 0.34 mm

### Diethyl (RS)-2-[(4-chlorophenyl)(5-methyl-3-oxo-2-phenyl-2,3-dihydro-1H-pyrazol-4-yl)methyl]propanedioate (II) Data collection

**Table:**

Oxford Diffraction Xcalibur with Sapphire CCD detector diffractometer	9574 independent reflections
Radiation source: Enhance (Mo) X-ray Source	6504 reflections with *I* > 2σ(*I*)
Graphite monochromator	*R*_int_ = 0.023
ω scans	θ_max_ = 26.1°, θ_min_ = 2.5°
Absorption correction: multi-scan (CrysAlis RED; Oxford Diffraction, 2009)	*h* = −15→16
*T*_min_ = 0.826, *T*_max_ = 0.936	*k* = −25→15
20936 measured reflections	*l* = −21→21

### Diethyl (RS)-2-[(4-chlorophenyl)(5-methyl-3-oxo-2-phenyl-2,3-dihydro-1H-pyrazol-4-yl)methyl]propanedioate (II) Refinement

**Table:**

Refinement on *F*^2^	Primary atom site location: difference Fourier map
Least-squares matrix: full	Hydrogen site location: mixed
*R*[*F*^2^ > 2σ(*F*^2^)] = 0.067	H atoms treated by a mixture of independent and constrained refinement
*wR*(*F*^2^) = 0.192	*w* = 1/[σ^2^(*F*_o_^2^) + (0.081*P*)^2^ + 4.3903*P*] where *P* = (*F*_o_^2^ + 2*F*_c_^2^)/3
*S* = 1.03	(Δ/σ)_max_ < 0.001
9574 reflections	Δρ_max_ = 1.06 e Å^−^^3^
746 parameters	Δρ_min_ = −0.91 e Å^−^^3^
571 restraints	

### Diethyl (RS)-2-[(4-chlorophenyl)(5-methyl-3-oxo-2-phenyl-2,3-dihydro-1H-pyrazol-4-yl)methyl]propanedioate (II) Special details

**Table:**

Geometry. All esds (except the esd in the dihedral angle between two l.s. planes) are estimated using the full covariance matrix. The cell esds are taken into account individually in the estimation of esds in distances, angles and torsion angles; correlations between esds in cell parameters are only used when they are defined by crystal symmetry. An approximate (isotropic) treatment of cell esds is used for estimating esds involving l.s. planes.

### Diethyl (RS)-2-[(4-chlorophenyl)(5-methyl-3-oxo-2-phenyl-2,3-dihydro-1H-pyrazol-4-yl)methyl]propanedioate (II) Fractional atomic coordinates and isotropic or equivalent isotropic displacement parameters (Å^2^)

**Table:**

	*x*	*y*	*z*	*U*_iso_*/*U*_eq_	Occ. (<1)
C11	0.6299 (3)	0.69693 (15)	0.54996 (16)	0.0459 (8)	
C12	0.6781 (2)	0.63001 (14)	0.56816 (14)	0.0356 (6)	
H12	0.7519	0.6342	0.5735	0.043*	
C13	0.6410 (2)	0.60627 (14)	0.64337 (15)	0.0381 (6)	
O11	0.54201 (19)	0.70614 (12)	0.54801 (13)	0.0599 (6)	
O12	0.69666 (19)	0.74133 (11)	0.53433 (14)	0.0622 (6)	
C14	0.6546 (4)	0.80629 (18)	0.5109 (2)	0.0787 (12)	
H14A	0.7057	0.8407	0.5219	0.094*	
H14B	0.5985	0.8164	0.5409	0.094*	
C15	0.6192 (5)	0.8084 (2)	0.4284 (3)	0.1003 (17)	
H15A	0.6004	0.8536	0.4139	0.150*	
H15B	0.5617	0.7793	0.4186	0.150*	
H15C	0.6724	0.7935	0.3986	0.150*	
O13	0.57468 (18)	0.56797 (13)	0.64923 (11)	0.0600 (7)	
O14	0.69144 (16)	0.63503 (11)	0.70316 (10)	0.0455 (5)	
C16	0.6597 (3)	0.61783 (18)	0.77903 (16)	0.0541 (9)	
H16A	0.6636	0.5695	0.7870	0.065*	
H16B	0.5903	0.6319	0.7824	0.065*	
C17	0.7268 (4)	0.6523 (3)	0.8379 (2)	0.0942 (16)	
H17A	0.7056	0.6431	0.8881	0.141*	
H17B	0.7243	0.6999	0.8285	0.141*	
H17C	0.7948	0.6366	0.8356	0.141*	
C121	0.6437 (2)	0.58366 (13)	0.50087 (14)	0.0330 (6)	
H121	0.5701	0.5799	0.4997	0.040*	
C131	0.6850 (2)	0.51432 (13)	0.50941 (14)	0.0342 (6)	
C132	0.6314 (2)	0.46401 (15)	0.47118 (16)	0.0404 (7)	
H132	0.5717	0.4742	0.4411	0.048*	
C133	0.6635 (3)	0.39972 (16)	0.47634 (18)	0.0491 (8)	
H133	0.6264	0.3658	0.4498	0.059*	
C134	0.7499 (3)	0.38485 (15)	0.5203 (2)	0.0531 (8)	
Cl14	0.79127 (10)	0.30382 (5)	0.52761 (8)	0.0914 (4)	
C135	0.8054 (3)	0.43380 (17)	0.5585 (2)	0.0605 (9)	
H135	0.8652	0.4232	0.5882	0.073*	
C136	0.7726 (2)	0.49824 (16)	0.55275 (18)	0.0500 (8)	
H136	0.8104	0.5321	0.5788	0.060*	
N141	0.6458 (2)	0.66271 (13)	0.31184 (14)	0.0421 (6)	
H141	0.621 (2)	0.6762 (16)	0.2702 (19)	0.051*	
C143	0.7619 (2)	0.63115 (12)	0.40440 (14)	0.0327 (6)	
O143	0.84662 (14)	0.62440 (9)	0.43944 (11)	0.0389 (5)	
C144	0.6658 (2)	0.61437 (13)	0.42527 (14)	0.0332 (6)	
C145	0.5972 (2)	0.63449 (14)	0.36709 (15)	0.0380 (6)	
C146	0.4864 (2)	0.62918 (19)	0.35895 (18)	0.0550 (9)	
H16C	0.4601	0.6463	0.4047	0.082*	
H16D	0.4672	0.5829	0.3519	0.082*	
H16E	0.4595	0.6549	0.3149	0.082*	
N142	0.7465 (2)	0.66035 (11)	0.33189 (12)	0.0384 (5)	0.635 (10)
C151	0.8086 (5)	0.6992 (5)	0.2879 (6)	0.0393 (14)	0.635 (10)
C152	0.9078 (5)	0.6817 (4)	0.2873 (5)	0.0496 (16)	0.635 (10)
H152	0.9323	0.6434	0.3135	0.059*	0.635 (10)
C153	0.9719 (6)	0.7207 (4)	0.2479 (4)	0.0553 (16)	0.635 (10)
H153	1.0403	0.7101	0.2497	0.066*	0.635 (10)
C154	0.9352 (6)	0.7740 (4)	0.2069 (4)	0.0521 (18)	0.635 (10)
H154	0.9776	0.7996	0.1788	0.062*	0.635 (10)
C155	0.8362 (6)	0.7904 (3)	0.2067 (3)	0.0462 (15)	0.635 (10)
H155	0.8114	0.8272	0.1776	0.055*	0.635 (10)
C156	0.7706 (6)	0.7539 (3)	0.2485 (4)	0.0398 (14)	0.635 (10)
H156	0.7033	0.7665	0.2495	0.048*	0.635 (10)
N162	0.7465 (2)	0.66035 (11)	0.33189 (12)	0.0384 (5)	0.365 (10)
C161	0.8264 (8)	0.6903 (9)	0.2947 (12)	0.042 (2)	0.365 (10)
C162	0.9227 (7)	0.6655 (7)	0.3048 (8)	0.047 (2)	0.365 (10)
H162	0.9375	0.6284	0.3366	0.057*	0.365 (10)
C163	0.9978 (8)	0.6961 (7)	0.2673 (7)	0.057 (2)	0.365 (10)
H163	1.0633	0.6793	0.2746	0.068*	0.365 (10)
C164	0.9788 (10)	0.7495 (7)	0.2205 (6)	0.055 (2)	0.365 (10)
H164	1.0302	0.7690	0.1952	0.067*	0.365 (10)
C165	0.8839 (11)	0.7743 (6)	0.2110 (7)	0.049 (2)	0.365 (10)
H165	0.8697	0.8113	0.1789	0.059*	0.365 (10)
C166	0.8073 (9)	0.7450 (6)	0.2486 (7)	0.046 (2)	0.365 (10)
H166	0.7423	0.7629	0.2422	0.055*	0.365 (10)
C21	0.3765 (5)	0.5785 (6)	0.1368 (4)	0.0489 (14)	0.690 (5)
C22	0.4883 (5)	0.5830 (6)	0.1422 (5)	0.0428 (13)	0.690 (5)
H22	0.5107	0.6107	0.1872	0.051*	0.690 (5)
C23	0.5284 (11)	0.5141 (6)	0.1560 (8)	0.0413 (14)	0.690 (5)
O21	0.3202 (6)	0.5833 (6)	0.0797 (4)	0.0532 (18)	0.690 (5)
O22	0.3455 (3)	0.5610 (2)	0.2056 (3)	0.0622 (11)	0.690 (5)
C24	0.2369 (5)	0.5524 (3)	0.2113 (5)	0.0811 (17)	0.690 (5)
H24A	0.2043	0.5356	0.1628	0.097*	0.690 (5)
H24B	0.2257	0.5204	0.2517	0.097*	0.690 (5)
C25	0.1953 (6)	0.6175 (4)	0.2290 (5)	0.107 (3)	0.690 (5)
H25A	0.1232	0.6138	0.2291	0.161*	0.690 (5)
H25B	0.2110	0.6496	0.1906	0.161*	0.690 (5)
H25C	0.2240	0.6321	0.2789	0.161*	0.690 (5)
O23	0.4928 (10)	0.4655 (7)	0.1240 (10)	0.050 (3)	0.690 (5)
O24	0.5936 (6)	0.5132 (5)	0.2171 (7)	0.0511 (16)	0.690 (5)
C26	0.6414 (5)	0.4503 (5)	0.2345 (6)	0.067 (2)	0.690 (5)
H26A	0.6482	0.4250	0.1876	0.080*	0.690 (5)
H26B	0.6025	0.4239	0.2682	0.080*	0.690 (5)
C27	0.7410 (4)	0.4669 (3)	0.2734 (4)	0.0725 (17)	0.690 (5)
H27A	0.7802	0.4901	0.2380	0.109*	0.690 (5)
H27B	0.7751	0.4262	0.2904	0.109*	0.690 (5)
H27C	0.7329	0.4951	0.3172	0.109*	0.690 (5)
C31	0.3853 (11)	0.5838 (15)	0.1230 (8)	0.053 (2)	0.310 (5)
C32	0.4961 (10)	0.5821 (13)	0.1417 (12)	0.044 (2)	0.310 (5)
H32	0.5140	0.6118	0.1859	0.053*	0.310 (5)
C33	0.530 (3)	0.5128 (14)	0.1626 (18)	0.043 (2)	0.310 (5)
O31	0.3383 (13)	0.5902 (14)	0.0615 (8)	0.052 (3)	0.310 (5)
O32	0.3418 (7)	0.5909 (6)	0.1899 (6)	0.069 (2)	0.310 (5)
C34	0.2316 (8)	0.5822 (9)	0.1838 (7)	0.077 (3)	0.310 (5)
H34A	0.1987	0.6234	0.1652	0.092*	0.310 (5)
H34B	0.2120	0.5465	0.1473	0.092*	0.310 (5)
C35	0.2008 (11)	0.5652 (10)	0.2594 (9)	0.098 (4)	0.310 (5)
H35A	0.1298	0.5551	0.2549	0.148*	0.310 (5)
H35B	0.2139	0.6027	0.2938	0.148*	0.310 (5)
H35C	0.2382	0.5268	0.2795	0.148*	0.310 (5)
O33	0.513 (2)	0.4664 (15)	0.120 (2)	0.044 (4)	0.310 (5)
O34	0.6086 (14)	0.5167 (11)	0.2132 (15)	0.052 (2)	0.310 (5)
C36	0.6519 (13)	0.4564 (12)	0.2451 (13)	0.062 (3)	0.310 (5)
H36A	0.6023	0.4204	0.2417	0.074*	0.310 (5)
H36B	0.6761	0.4629	0.2990	0.074*	0.310 (5)
C37	0.7360 (10)	0.4398 (7)	0.1997 (9)	0.078 (4)	0.310 (5)
H37A	0.7790	0.4783	0.1971	0.117*	0.310 (5)
H37B	0.7100	0.4269	0.1484	0.117*	0.310 (5)
H37C	0.7742	0.4032	0.2239	0.117*	0.310 (5)
C221	0.5336 (2)	0.61330 (14)	0.07027 (16)	0.0432 (7)	
H221	0.4996	0.5914	0.0245	0.052*	
C231	0.6454 (2)	0.60192 (14)	0.06837 (14)	0.0382 (6)	
C232	0.6791 (2)	0.54388 (15)	0.03788 (18)	0.0487 (8)	
H232	0.6321	0.5130	0.0160	0.058*	
C233	0.7794 (3)	0.52960 (18)	0.0384 (2)	0.0639 (10)	
H233	0.8005	0.4899	0.0166	0.077*	
C234	0.8482 (3)	0.57381 (19)	0.0711 (2)	0.0619 (10)	
Cl24	0.97561 (8)	0.55516 (7)	0.07838 (8)	0.0994 (4)	
C235	0.8165 (3)	0.63321 (19)	0.0982 (2)	0.0678 (11)	
H235	0.8636	0.6648	0.1182	0.081*	
C236	0.7156 (3)	0.64689 (16)	0.09625 (18)	0.0530 (8)	
H236	0.6948	0.6882	0.1146	0.064*	
N241	0.4525 (2)	0.78135 (12)	0.01862 (14)	0.0422 (6)	
H241	0.414 (2)	0.8097 (17)	−0.0039 (18)	0.051*	
N242	0.47596 (19)	0.79052 (11)	0.09604 (13)	0.0415 (6)	
C243	0.5118 (3)	0.73182 (14)	0.12740 (17)	0.0467 (8)	
O243	0.5437 (2)	0.72724 (11)	0.19701 (12)	0.0633 (7)	
C244	0.5044 (2)	0.68516 (14)	0.06668 (16)	0.0426 (7)	
C245	0.4653 (2)	0.71757 (14)	0.00195 (16)	0.0406 (7)	
C246	0.4373 (3)	0.69103 (17)	−0.07580 (18)	0.0600 (9)	
H26C	0.4410	0.6428	−0.0747	0.090*	
H26D	0.4830	0.7082	−0.1107	0.090*	
H26E	0.3696	0.7047	−0.0929	0.090*	
C251	0.4870 (2)	0.85489 (14)	0.12742 (17)	0.0428 (7)	
C252	0.4658 (3)	0.86487 (16)	0.2030 (2)	0.0566 (9)	
H252	0.4430	0.8294	0.2319	0.068*	
C253	0.4787 (3)	0.92726 (18)	0.2345 (2)	0.0700 (11)	
H253	0.4646	0.9350	0.2853	0.084*	
C254	0.5124 (3)	0.97799 (17)	0.1917 (3)	0.0727 (12)	
H254	0.5223	1.0205	0.2136	0.087*	
C255	0.5319 (3)	0.96767 (17)	0.1170 (2)	0.0660 (10)	
H255	0.5536	1.0034	0.0881	0.079*	
C256	0.5199 (2)	0.90593 (15)	0.08439 (19)	0.0501 (8)	
H256	0.5340	0.8987	0.0336	0.060*	

### Diethyl (RS)-2-[(4-chlorophenyl)(5-methyl-3-oxo-2-phenyl-2,3-dihydro-1H-pyrazol-4-yl)methyl]propanedioate (II) Atomic displacement parameters (Å^2^)

**Table:**

	*U*^11^	*U*^22^	*U*^33^	*U*^12^	*U*^13^	*U*^23^
C11	0.067 (2)	0.0441 (17)	0.0285 (15)	−0.0069 (16)	0.0139 (14)	−0.0047 (12)
C12	0.0369 (15)	0.0398 (15)	0.0304 (14)	−0.0055 (12)	0.0047 (11)	−0.0004 (11)
C13	0.0421 (16)	0.0437 (16)	0.0281 (14)	−0.0043 (14)	0.0019 (12)	−0.0013 (12)
O11	0.0631 (17)	0.0569 (15)	0.0610 (15)	0.0124 (12)	0.0132 (12)	0.0012 (11)
O12	0.0735 (17)	0.0434 (13)	0.0701 (16)	−0.0092 (12)	0.0089 (13)	−0.0005 (11)
C14	0.111 (4)	0.040 (2)	0.084 (3)	−0.006 (2)	0.002 (3)	−0.0002 (19)
C15	0.157 (5)	0.060 (3)	0.080 (3)	0.008 (3)	−0.009 (3)	0.014 (2)
O13	0.0606 (15)	0.0857 (18)	0.0338 (11)	−0.0324 (14)	0.0053 (10)	0.0000 (11)
O14	0.0550 (13)	0.0546 (13)	0.0267 (10)	−0.0104 (11)	0.0028 (9)	−0.0021 (9)
C16	0.070 (2)	0.066 (2)	0.0264 (15)	−0.0015 (18)	0.0065 (14)	0.0042 (14)
C17	0.117 (4)	0.133 (4)	0.0321 (19)	−0.033 (3)	0.003 (2)	−0.013 (2)
C121	0.0325 (14)	0.0389 (15)	0.0269 (13)	−0.0060 (12)	−0.0008 (10)	0.0010 (11)
C131	0.0390 (15)	0.0367 (14)	0.0273 (13)	−0.0085 (12)	0.0063 (11)	0.0033 (11)
C132	0.0407 (16)	0.0460 (17)	0.0352 (15)	−0.0098 (14)	0.0071 (12)	−0.0037 (12)
C133	0.058 (2)	0.0435 (17)	0.0481 (18)	−0.0174 (16)	0.0184 (15)	−0.0112 (14)
C134	0.065 (2)	0.0341 (16)	0.063 (2)	−0.0011 (15)	0.0187 (18)	0.0026 (14)
Cl14	0.1046 (9)	0.0378 (5)	0.1346 (10)	0.0086 (5)	0.0256 (7)	0.0048 (5)
C135	0.058 (2)	0.0471 (19)	0.073 (2)	0.0044 (17)	−0.0104 (18)	0.0070 (17)
C136	0.0497 (19)	0.0413 (17)	0.0557 (19)	−0.0072 (15)	−0.0125 (15)	0.0013 (14)
N141	0.0496 (16)	0.0474 (15)	0.0280 (12)	−0.0056 (12)	−0.0038 (11)	0.0079 (11)
C143	0.0435 (16)	0.0249 (12)	0.0296 (13)	−0.0070 (12)	0.0017 (11)	−0.0036 (10)
O143	0.0369 (11)	0.0362 (10)	0.0427 (11)	−0.0054 (9)	−0.0009 (9)	−0.0050 (8)
C144	0.0374 (15)	0.0334 (14)	0.0284 (13)	−0.0056 (12)	0.0016 (11)	−0.0006 (11)
C145	0.0457 (17)	0.0406 (15)	0.0271 (13)	−0.0025 (13)	0.0007 (12)	0.0021 (11)
C146	0.0414 (18)	0.078 (2)	0.0438 (18)	−0.0009 (17)	−0.0055 (14)	0.0095 (16)
N142	0.0508 (14)	0.0337 (12)	0.0313 (11)	−0.0104 (11)	0.0071 (10)	0.0012 (9)
C151	0.058 (3)	0.034 (3)	0.027 (3)	−0.018 (2)	0.010 (3)	−0.007 (2)
C152	0.066 (3)	0.043 (3)	0.041 (3)	−0.014 (3)	0.017 (3)	0.002 (2)
C153	0.067 (3)	0.050 (4)	0.052 (4)	−0.016 (3)	0.024 (3)	0.003 (3)
C154	0.067 (4)	0.050 (3)	0.042 (3)	−0.021 (3)	0.025 (3)	−0.005 (2)
C155	0.068 (4)	0.039 (3)	0.032 (2)	−0.019 (3)	0.011 (3)	−0.004 (2)
C156	0.058 (3)	0.033 (2)	0.030 (2)	−0.014 (3)	0.010 (3)	−0.0049 (19)
N162	0.0508 (14)	0.0337 (12)	0.0313 (11)	−0.0104 (11)	0.0071 (10)	0.0012 (9)
C161	0.062 (4)	0.035 (4)	0.030 (3)	−0.018 (3)	0.011 (4)	0.000 (3)
C162	0.062 (4)	0.047 (5)	0.036 (4)	−0.020 (4)	0.019 (4)	0.004 (3)
C163	0.070 (5)	0.057 (5)	0.045 (4)	−0.019 (4)	0.014 (4)	0.008 (4)
C164	0.075 (5)	0.051 (5)	0.042 (4)	−0.021 (4)	0.015 (4)	0.004 (4)
C165	0.068 (5)	0.044 (4)	0.038 (3)	−0.018 (4)	0.016 (4)	−0.001 (3)
C166	0.067 (4)	0.039 (4)	0.030 (3)	−0.016 (4)	0.006 (4)	−0.006 (3)
C21	0.045 (3)	0.039 (3)	0.063 (3)	0.006 (2)	0.007 (2)	−0.006 (3)
C22	0.048 (3)	0.033 (2)	0.047 (2)	0.002 (2)	−0.001 (2)	−0.002 (2)
C23	0.043 (2)	0.033 (2)	0.047 (3)	−0.001 (2)	0.007 (2)	0.007 (2)
O21	0.045 (4)	0.046 (3)	0.067 (3)	−0.001 (3)	−0.001 (3)	−0.005 (3)
O22	0.056 (2)	0.057 (2)	0.077 (3)	0.004 (2)	0.0188 (18)	0.007 (2)
C24	0.074 (3)	0.077 (4)	0.097 (4)	0.012 (3)	0.026 (3)	0.019 (3)
C25	0.110 (6)	0.103 (6)	0.112 (6)	0.031 (5)	0.025 (5)	0.015 (5)
O23	0.054 (6)	0.035 (3)	0.059 (4)	−0.004 (3)	0.002 (4)	0.001 (2)
O24	0.049 (3)	0.042 (2)	0.060 (2)	−0.002 (2)	−0.006 (2)	0.007 (2)
C26	0.060 (3)	0.054 (3)	0.083 (4)	0.005 (3)	−0.019 (3)	0.018 (3)
C27	0.055 (3)	0.081 (4)	0.078 (4)	0.006 (3)	−0.013 (3)	0.012 (3)
C31	0.050 (4)	0.046 (4)	0.065 (4)	0.001 (4)	0.011 (4)	−0.006 (4)
C32	0.043 (4)	0.036 (4)	0.052 (4)	0.001 (4)	0.003 (4)	−0.001 (4)
C33	0.047 (4)	0.033 (4)	0.050 (4)	0.002 (4)	0.005 (4)	0.006 (4)
O31	0.033 (5)	0.043 (6)	0.081 (7)	−0.005 (4)	0.018 (5)	−0.005 (6)
O32	0.062 (4)	0.068 (4)	0.080 (4)	0.019 (4)	0.015 (3)	0.027 (4)
C34	0.061 (5)	0.082 (5)	0.092 (5)	0.020 (5)	0.031 (5)	0.026 (5)
C35	0.078 (8)	0.108 (9)	0.114 (9)	0.019 (8)	0.038 (7)	0.029 (8)
O33	0.044 (9)	0.031 (6)	0.056 (7)	0.006 (6)	0.006 (7)	0.001 (5)
O34	0.050 (4)	0.042 (4)	0.062 (4)	0.001 (4)	−0.004 (4)	0.015 (4)
C36	0.061 (5)	0.049 (5)	0.072 (5)	0.007 (5)	−0.008 (5)	0.014 (5)
C37	0.065 (7)	0.070 (7)	0.098 (8)	0.003 (6)	−0.002 (6)	0.024 (6)
C221	0.060 (2)	0.0305 (14)	0.0377 (15)	0.0052 (14)	−0.0051 (13)	0.0015 (12)
C231	0.0551 (18)	0.0335 (14)	0.0253 (13)	0.0030 (13)	−0.0003 (12)	0.0014 (11)
C232	0.053 (2)	0.0391 (17)	0.0537 (19)	0.0002 (15)	0.0025 (15)	−0.0101 (14)
C233	0.064 (2)	0.0471 (19)	0.082 (3)	0.0014 (18)	0.0125 (19)	−0.0251 (18)
C234	0.056 (2)	0.063 (2)	0.068 (2)	−0.0020 (18)	0.0105 (17)	−0.0196 (18)
Cl24	0.0581 (6)	0.1094 (10)	0.1321 (11)	−0.0003 (6)	0.0165 (6)	−0.0429 (8)
C235	0.065 (2)	0.067 (2)	0.072 (2)	−0.016 (2)	0.0119 (19)	−0.036 (2)
C236	0.061 (2)	0.0464 (18)	0.0516 (19)	−0.0005 (16)	0.0072 (16)	−0.0193 (15)
N241	0.0477 (15)	0.0354 (13)	0.0418 (14)	0.0095 (12)	−0.0059 (11)	0.0069 (11)
N242	0.0531 (15)	0.0313 (12)	0.0387 (13)	0.0080 (11)	−0.0036 (11)	0.0047 (10)
C243	0.063 (2)	0.0352 (16)	0.0402 (17)	0.0084 (15)	−0.0036 (14)	0.0067 (13)
O243	0.108 (2)	0.0385 (12)	0.0393 (12)	0.0141 (13)	−0.0158 (12)	0.0024 (9)
C244	0.0521 (18)	0.0320 (15)	0.0418 (16)	0.0072 (13)	−0.0052 (13)	0.0035 (12)
C245	0.0445 (17)	0.0343 (15)	0.0423 (16)	0.0035 (13)	−0.0001 (13)	0.0034 (12)
C246	0.084 (3)	0.0509 (19)	0.0417 (18)	0.0134 (19)	−0.0102 (17)	0.0010 (15)
C251	0.0422 (17)	0.0319 (15)	0.0525 (18)	0.0040 (13)	−0.0049 (13)	0.0026 (13)
C252	0.069 (2)	0.0394 (17)	0.062 (2)	0.0019 (16)	0.0071 (17)	−0.0016 (15)
C253	0.089 (3)	0.052 (2)	0.069 (2)	0.008 (2)	0.001 (2)	−0.0145 (18)
C254	0.082 (3)	0.0353 (19)	0.095 (3)	−0.0028 (18)	−0.022 (2)	−0.0069 (19)
C255	0.069 (2)	0.0418 (19)	0.082 (3)	−0.0062 (18)	−0.017 (2)	0.0112 (18)
C256	0.0509 (19)	0.0386 (17)	0.058 (2)	−0.0001 (15)	−0.0088 (15)	0.0085 (14)

### Diethyl (RS)-2-[(4-chlorophenyl)(5-methyl-3-oxo-2-phenyl-2,3-dihydro-1H-pyrazol-4-yl)methyl]propanedioate (II) Geometric parameters (Å, º)

**Table:**

C11—O11	1.204 (4)	C23—O23	1.215 (6)
C11—O12	1.324 (4)	C23—O24	1.332 (6)
C11—C12	1.527 (4)	O22—C24	1.496 (6)
C12—C13	1.545 (4)	C24—C25	1.480 (7)
C12—C121	1.556 (4)	C24—H24A	0.9900
C12—H12	1.0000	C24—H24B	0.9900
C13—O13	1.201 (3)	C25—H25A	0.9800
C13—O14	1.340 (3)	C25—H25B	0.9800
O12—C14	1.480 (4)	C25—H25C	0.9800
C14—C15	1.497 (6)	O24—C26	1.452 (5)
C14—H14A	0.9900	C26—C27	1.496 (9)
C14—H14B	0.9900	C26—H26A	0.9900
C15—H15A	0.9800	C26—H26B	0.9900
C15—H15B	0.9800	C27—H27A	0.9800
C15—H15C	0.9800	C27—H27B	0.9800
O14—C16	1.492 (3)	C27—H27C	0.9800
C16—C17	1.493 (5)	C31—O31	1.217 (8)
C16—H16A	0.9900	C31—O32	1.382 (9)
C16—H16B	0.9900	C31—C32	1.508 (8)
C17—H17A	0.9800	C32—C33	1.513 (9)
C17—H17B	0.9800	C32—C221	1.543 (13)
C17—H17C	0.9800	C32—H32	1.0000
C121—C131	1.516 (4)	C33—O33	1.219 (10)
C121—C144	1.533 (3)	C33—O34	1.334 (9)
C121—H121	1.0000	O32—C34	1.499 (8)
C131—C132	1.392 (4)	C34—C35	1.482 (9)
C131—C136	1.392 (4)	C34—H34A	0.9900
C132—C133	1.375 (4)	C34—H34B	0.9900
C132—H132	0.9500	C35—H35A	0.9800
C133—C134	1.378 (5)	C35—H35B	0.9800
C133—H133	0.9500	C35—H35C	0.9800
C134—C135	1.384 (5)	O34—C36	1.450 (8)
C134—Cl14	1.737 (3)	C36—C37	1.494 (11)
C135—C136	1.381 (5)	C36—H36A	0.9900
C135—H135	0.9500	C36—H36B	0.9900
C136—H136	0.9500	C37—H37A	0.9800
N141—C145	1.357 (4)	C37—H37B	0.9800
N141—N142	1.379 (3)	C37—H37C	0.9800
N141—H141	0.83 (3)	C221—C244	1.510 (4)
C143—O143	1.261 (3)	C221—C231	1.537 (4)
C143—N142	1.413 (3)	C221—H221	1.0000
C143—C144	1.429 (4)	C231—C236	1.376 (4)
C144—C145	1.384 (4)	C231—C232	1.390 (4)
C145—C146	1.500 (4)	C232—C233	1.390 (5)
C146—H16C	0.9800	C232—H232	0.9500
C146—H16D	0.9800	C233—C234	1.381 (5)
C146—H16E	0.9800	C233—H233	0.9500
N142—C151	1.436 (5)	C234—C235	1.380 (5)
C151—C156	1.385 (6)	C234—Cl24	1.762 (4)
C151—C152	1.391 (7)	C235—C236	1.394 (5)
C152—C153	1.408 (7)	C235—H235	0.9500
C152—H152	0.9500	C236—H236	0.9500
C153—C154	1.370 (9)	N241—C245	1.342 (4)
C153—H153	0.9500	N241—N242	1.392 (3)
C154—C155	1.382 (9)	N241—H241	0.85 (3)
C154—H154	0.9500	N242—C243	1.383 (4)
C155—C156	1.418 (7)	N242—C251	1.422 (4)
C155—H155	0.9500	C243—O243	1.272 (3)
C156—H156	0.9500	C243—C244	1.430 (4)
C161—C166	1.387 (8)	C244—C245	1.384 (4)
C161—C162	1.396 (9)	C245—C246	1.495 (4)
C162—C163	1.412 (9)	C246—H26C	0.9800
C162—H162	0.9500	C246—H26D	0.9800
C163—C164	1.373 (11)	C246—H26E	0.9800
C163—H163	0.9500	C251—C256	1.384 (4)
C164—C165	1.378 (12)	C251—C252	1.411 (5)
C164—H164	0.9500	C252—C253	1.388 (5)
C165—C166	1.417 (9)	C252—H252	0.9500
C165—H165	0.9500	C253—C254	1.381 (6)
C166—H166	0.9500	C253—H253	0.9500
C21—O21	1.214 (5)	C254—C255	1.391 (6)
C21—O22	1.372 (6)	C254—H254	0.9500
C21—C22	1.513 (6)	C255—C256	1.382 (5)
C22—C23	1.511 (5)	C255—H255	0.9500
C22—C221	1.590 (7)	C256—H256	0.9500
C22—H22	1.0000		
			
O11—C11—O12	125.6 (3)	C21—O22—C24	118.4 (5)
O11—C11—C12	123.1 (3)	C25—C24—O22	107.9 (6)
O12—C11—C12	111.2 (3)	C25—C24—H24A	110.1
C11—C12—C13	107.0 (2)	O22—C24—H24A	110.1
C11—C12—C121	106.5 (2)	C25—C24—H24B	110.1
C13—C12—C121	112.0 (2)	O22—C24—H24B	110.1
C11—C12—H12	110.4	H24A—C24—H24B	108.4
C13—C12—H12	110.4	C24—C25—H25A	109.5
C121—C12—H12	110.4	C24—C25—H25B	109.5
O13—C13—O14	123.0 (2)	H25A—C25—H25B	109.5
O13—C13—C12	125.7 (2)	C24—C25—H25C	109.5
O14—C13—C12	111.3 (2)	H25A—C25—H25C	109.5
C11—O12—C14	114.3 (3)	H25B—C25—H25C	109.5
O12—C14—C15	112.3 (3)	C23—O24—C26	115.7 (6)
O12—C14—H14A	109.1	O24—C26—C27	105.5 (6)
C15—C14—H14A	109.1	O24—C26—H26A	110.6
O12—C14—H14B	109.1	C27—C26—H26A	110.6
C15—C14—H14B	109.1	O24—C26—H26B	110.6
H14A—C14—H14B	107.9	C27—C26—H26B	110.6
C14—C15—H15A	109.5	H26A—C26—H26B	108.8
C14—C15—H15B	109.5	C26—C27—H27A	109.5
H15A—C15—H15B	109.5	C26—C27—H27B	109.5
C14—C15—H15C	109.5	H27A—C27—H27B	109.5
H15A—C15—H15C	109.5	C26—C27—H27C	109.5
H15B—C15—H15C	109.5	H27A—C27—H27C	109.5
C13—O14—C16	116.3 (2)	H27B—C27—H27C	109.5
O14—C16—C17	108.2 (3)	O31—C31—O32	121.9 (11)
O14—C16—H16A	110.1	O31—C31—C32	128.9 (11)
C17—C16—H16A	110.1	O32—C31—C32	108.0 (9)
O14—C16—H16B	110.1	C31—C32—C33	110.2 (11)
C17—C16—H16B	110.1	C31—C32—C221	102.0 (10)
H16A—C16—H16B	108.4	C33—C32—C221	118 (2)
C16—C17—H17A	109.5	C31—C32—H32	108.8
C16—C17—H17B	109.5	C33—C32—H32	108.8
H17A—C17—H17B	109.5	C221—C32—H32	108.8
C16—C17—H17C	109.5	O33—C33—O34	124.0 (16)
H17A—C17—H17C	109.5	O33—C33—C32	122.0 (16)
H17B—C17—H17C	109.5	O34—C33—C32	108.2 (11)
C131—C121—C144	111.3 (2)	C31—O32—C34	115.3 (9)
C131—C121—C12	113.7 (2)	C35—C34—O32	109.0 (9)
C144—C121—C12	110.9 (2)	C35—C34—H34A	109.9
C131—C121—H121	106.8	O32—C34—H34A	109.9
C144—C121—H121	106.8	C35—C34—H34B	109.9
C12—C121—H121	106.8	O32—C34—H34B	109.9
C132—C131—C136	118.5 (3)	H34A—C34—H34B	108.3
C132—C131—C121	117.4 (3)	C34—C35—H35A	109.5
C136—C131—C121	124.1 (2)	C34—C35—H35B	109.5
C133—C132—C131	121.0 (3)	H35A—C35—H35B	109.5
C133—C132—H132	119.5	C34—C35—H35C	109.5
C131—C132—H132	119.5	H35A—C35—H35C	109.5
C132—C133—C134	119.5 (3)	H35B—C35—H35C	109.5
C132—C133—H133	120.3	C33—O34—C36	118.9 (13)
C134—C133—H133	120.3	O34—C36—C37	106.4 (12)
C133—C134—C135	120.9 (3)	O34—C36—H36A	110.4
C133—C134—Cl14	120.1 (3)	C37—C36—H36A	110.4
C135—C134—Cl14	119.0 (3)	O34—C36—H36B	110.4
C136—C135—C134	119.1 (3)	C37—C36—H36B	110.4
C136—C135—H135	120.4	H36A—C36—H36B	108.6
C134—C135—H135	120.4	C36—C37—H37A	109.5
C135—C136—C131	121.0 (3)	C36—C37—H37B	109.5
C135—C136—H136	119.5	H37A—C37—H37B	109.5
C131—C136—H136	119.5	C36—C37—H37C	109.5
C145—N141—N142	109.6 (2)	H37A—C37—H37C	109.5
C145—N141—H141	127 (2)	H37B—C37—H37C	109.5
N142—N141—H141	123 (2)	C244—C221—C231	113.5 (3)
O143—C143—N142	123.0 (2)	C244—C221—C32	108.9 (10)
O143—C143—C144	131.1 (2)	C231—C221—C32	111.0 (5)
N142—C143—C144	105.9 (2)	C244—C221—C22	106.7 (5)
C145—C144—C143	107.6 (2)	C231—C221—C22	114.5 (3)
C145—C144—C121	126.8 (2)	C244—C221—H221	107.2
C143—C144—C121	125.5 (2)	C231—C221—H221	107.2
N141—C145—C144	108.9 (3)	C22—C221—H221	107.2
N141—C145—C146	120.7 (3)	C236—C231—C232	117.3 (3)
C144—C145—C146	130.4 (3)	C236—C231—C221	122.9 (3)
C145—C146—H16C	109.5	C232—C231—C221	119.8 (3)
C145—C146—H16D	109.5	C233—C232—C231	122.1 (3)
H16C—C146—H16D	109.5	C233—C232—H232	119.0
C145—C146—H16E	109.5	C231—C232—H232	119.0
H16C—C146—H16E	109.5	C234—C233—C232	119.3 (3)
H16D—C146—H16E	109.5	C234—C233—H233	120.4
N141—N142—C143	107.9 (2)	C232—C233—H233	120.4
N141—N142—C151	117.3 (4)	C235—C234—C233	119.6 (3)
C143—N142—C151	132.8 (4)	C235—C234—Cl24	119.8 (3)
C156—C151—C152	121.0 (4)	C233—C234—Cl24	120.7 (3)
C156—C151—N142	120.2 (5)	C234—C235—C236	120.1 (3)
C152—C151—N142	118.8 (5)	C234—C235—H235	119.9
C151—C152—C153	120.2 (6)	C236—C235—H235	119.9
C151—C152—H152	119.9	C231—C236—C235	121.5 (3)
C153—C152—H152	119.9	C231—C236—H236	119.3
C154—C153—C152	119.8 (6)	C235—C236—H236	119.3
C154—C153—H153	120.1	C245—N241—N242	108.9 (2)
C152—C153—H153	120.1	C245—N241—H241	129 (2)
C153—C154—C155	119.7 (5)	N242—N241—H241	117 (2)
C153—C154—H154	120.1	C243—N242—N241	108.7 (2)
C155—C154—H154	120.1	C243—N242—C251	127.8 (2)
C154—C155—C156	122.0 (6)	N241—N242—C251	121.0 (2)
C154—C155—H155	119.0	O243—C243—N242	121.8 (3)
C156—C155—H155	119.0	O243—C243—C244	132.5 (3)
C151—C156—C155	117.4 (6)	N242—C243—C244	105.6 (2)
C151—C156—H156	121.3	C245—C244—C243	107.7 (2)
C155—C156—H156	121.3	C245—C244—C221	124.9 (3)
C166—C161—C162	119.0 (7)	C243—C244—C221	127.4 (3)
C161—C162—C163	119.2 (9)	N241—C245—C244	108.8 (3)
C161—C162—H162	120.4	N241—C245—C246	121.5 (3)
C163—C162—H162	120.4	C244—C245—C246	129.6 (3)
C164—C163—C162	122.0 (9)	C245—C246—H26C	109.5
C164—C163—H163	119.0	C245—C246—H26D	109.5
C162—C163—H163	119.0	H26C—C246—H26D	109.5
C163—C164—C165	118.8 (8)	C245—C246—H26E	109.5
C163—C164—H164	120.6	H26C—C246—H26E	109.5
C165—C164—H164	120.6	H26D—C246—H26E	109.5
C164—C165—C166	120.4 (9)	C256—C251—C252	121.4 (3)
C164—C165—H165	119.8	C256—C251—N242	119.9 (3)
C166—C165—H165	119.8	C252—C251—N242	118.7 (3)
C161—C166—C165	120.5 (9)	C253—C252—C251	118.9 (3)
C161—C166—H166	119.7	C253—C252—H252	120.5
C165—C166—H166	119.7	C251—C252—H252	120.5
O21—C21—O22	122.8 (6)	C254—C253—C252	119.6 (4)
O21—C21—C22	126.6 (6)	C254—C253—H253	120.2
O22—C21—C22	110.3 (4)	C252—C253—H253	120.2
C23—C22—C21	107.3 (5)	C253—C254—C255	120.9 (3)
C23—C22—C221	109.1 (10)	C253—C254—H254	119.6
C21—C22—C221	115.6 (5)	C255—C254—H254	119.6
C23—C22—H22	108.2	C256—C255—C254	120.6 (3)
C21—C22—H22	108.2	C256—C255—H255	119.7
C221—C22—H22	108.2	C254—C255—H255	119.7
O23—C23—O24	125.1 (7)	C255—C256—C251	118.6 (3)
O23—C23—C22	123.5 (6)	C255—C256—H256	120.7
O24—C23—C22	110.2 (5)	C251—C256—H256	120.7
			
O11—C11—C12—C13	−52.5 (4)	C21—O22—C24—C25	−87.7 (9)
O12—C11—C12—C13	129.8 (2)	O23—C23—O24—C26	15 (3)
O11—C11—C12—C121	67.4 (3)	C22—C23—O24—C26	−177.1 (9)
O12—C11—C12—C121	−110.3 (3)	C23—O24—C26—C27	150.7 (13)
C11—C12—C13—O13	98.4 (4)	O31—C31—C32—C33	−108 (3)
C121—C12—C13—O13	−17.9 (4)	O32—C31—C32—C33	85 (3)
C11—C12—C13—O14	−80.3 (3)	O31—C31—C32—C221	18 (4)
C121—C12—C13—O14	163.3 (2)	O32—C31—C32—C221	−149 (2)
O11—C11—O12—C14	−1.7 (4)	C31—C32—C33—O33	58 (5)
C12—C11—O12—C14	175.9 (3)	C221—C32—C33—O33	−59 (4)
C11—O12—C14—C15	−83.5 (5)	C31—C32—C33—O34	−148 (2)
O13—C13—O14—C16	−1.0 (4)	C221—C32—C33—O34	95 (3)
C12—C13—O14—C16	177.8 (2)	O31—C31—O32—C34	21 (3)
C13—O14—C16—C17	177.7 (3)	C32—C31—O32—C34	−170.1 (15)
C11—C12—C121—C131	180.0 (2)	C31—O32—C34—C35	158.2 (19)
C13—C12—C121—C131	−63.4 (3)	O33—C33—O34—C36	−29 (6)
C11—C12—C121—C144	53.6 (3)	C32—C33—O34—C36	177 (2)
C13—C12—C121—C144	170.2 (2)	C33—O34—C36—C37	96 (3)
C144—C121—C131—C132	−78.9 (3)	C31—C32—C221—C244	67.7 (17)
C12—C121—C131—C132	155.0 (2)	C33—C32—C221—C244	−171.5 (10)
C144—C121—C131—C136	101.0 (3)	C31—C32—C221—C231	−166.6 (13)
C12—C121—C131—C136	−25.1 (4)	C33—C32—C221—C231	−45.8 (15)
C136—C131—C132—C133	0.5 (4)	C31—C32—C221—C22	16 (25)
C121—C131—C132—C133	−179.6 (2)	C33—C32—C221—C22	136 (27)
C131—C132—C133—C134	0.2 (4)	C23—C22—C221—C244	−171.1 (4)
C132—C133—C134—C135	−0.8 (5)	C21—C22—C221—C244	67.8 (9)
C132—C133—C134—Cl14	179.8 (2)	C23—C22—C221—C231	−44.6 (7)
C133—C134—C135—C136	0.6 (5)	C21—C22—C221—C231	−165.7 (6)
Cl14—C134—C135—C136	−180.0 (3)	C23—C22—C221—C32	−42 (26)
C134—C135—C136—C131	0.1 (5)	C21—C22—C221—C32	−163 (27)
C132—C131—C136—C135	−0.6 (5)	C244—C221—C231—C236	29.3 (4)
C121—C131—C136—C135	179.5 (3)	C32—C221—C231—C236	−93.8 (13)
O143—C143—C144—C145	177.9 (3)	C22—C221—C231—C236	−93.7 (6)
N142—C143—C144—C145	−1.1 (3)	C244—C221—C231—C232	−151.1 (3)
O143—C143—C144—C121	1.4 (5)	C32—C221—C231—C232	85.8 (13)
N142—C143—C144—C121	−177.6 (2)	C22—C221—C231—C232	85.9 (6)
C131—C121—C144—C145	117.6 (3)	C236—C231—C232—C233	3.1 (5)
C12—C121—C144—C145	−114.7 (3)	C221—C231—C232—C233	−176.5 (3)
C131—C121—C144—C143	−66.6 (3)	C231—C232—C233—C234	0.9 (6)
C12—C121—C144—C143	61.1 (3)	C232—C233—C234—C235	−4.2 (6)
N142—N141—C145—C144	1.3 (3)	C232—C233—C234—Cl24	175.9 (3)
N142—N141—C145—C146	−178.7 (3)	C233—C234—C235—C236	3.5 (6)
C143—C144—C145—N141	−0.1 (3)	Cl24—C234—C235—C236	−176.7 (3)
C121—C144—C145—N141	176.3 (2)	C232—C231—C236—C235	−3.9 (5)
C143—C144—C145—C146	179.9 (3)	C221—C231—C236—C235	175.7 (3)
C121—C144—C145—C146	−3.7 (5)	C234—C235—C236—C231	0.7 (6)
C145—N141—N142—C143	−2.0 (3)	C245—N241—N242—C243	4.8 (3)
C145—N141—N142—C151	−168.0 (6)	C245—N241—N242—C251	168.2 (3)
O143—C143—N142—N141	−177.2 (2)	N241—N242—C243—O243	176.3 (3)
C144—C143—N142—N141	1.8 (3)	C251—N242—C243—O243	14.5 (5)
O143—C143—N142—C151	−14.2 (7)	N241—N242—C243—C244	−3.1 (3)
C144—C143—N142—C151	164.8 (6)	C251—N242—C243—C244	−165.0 (3)
N141—N142—C151—C156	24.1 (12)	O243—C243—C244—C245	−179.0 (4)
C143—N142—C151—C156	−137.7 (7)	N242—C243—C244—C245	0.3 (4)
N141—N142—C151—C152	−157.6 (7)	O243—C243—C244—C221	0.8 (6)
C143—N142—C151—C152	40.6 (13)	N242—C243—C244—C221	−179.9 (3)
C156—C151—C152—C153	1.6 (14)	C231—C221—C244—C245	99.7 (4)
N142—C151—C152—C153	−176.7 (8)	C32—C221—C244—C245	−136.1 (9)
C151—C152—C153—C154	−3.3 (11)	C22—C221—C244—C245	−133.1 (5)
C152—C153—C154—C155	2.2 (10)	C231—C221—C244—C243	−80.0 (4)
C153—C154—C155—C156	0.7 (9)	C32—C221—C244—C243	44.2 (9)
C152—C151—C156—C155	1.2 (13)	C22—C221—C244—C243	47.2 (5)
N142—C151—C156—C155	179.5 (8)	N242—N241—C245—C244	−4.6 (3)
C154—C155—C156—C151	−2.4 (10)	N242—N241—C245—C246	174.4 (3)
C166—C161—C162—C163	−1 (3)	C243—C244—C245—N241	2.6 (4)
C161—C162—C163—C164	−1 (2)	C221—C244—C245—N241	−177.1 (3)
C162—C163—C164—C165	1.0 (18)	C243—C244—C245—C246	−176.2 (3)
C163—C164—C165—C166	−0.2 (19)	C221—C244—C245—C246	4.0 (6)
C162—C161—C166—C165	1 (3)	C243—N242—C251—C256	130.0 (3)
C164—C165—C166—C161	−1 (2)	N241—N242—C251—C256	−30.0 (4)
O21—C21—C22—C23	−106.7 (12)	C243—N242—C251—C252	−48.7 (5)
O22—C21—C22—C23	67.0 (12)	N241—N242—C251—C252	151.4 (3)
O21—C21—C22—C221	15.3 (15)	C256—C251—C252—C253	−0.2 (5)
O22—C21—C22—C221	−171.0 (9)	N242—C251—C252—C253	178.4 (3)
C21—C22—C23—O23	42 (2)	C251—C252—C253—C254	−0.3 (6)
C221—C22—C23—O23	−84.4 (18)	C252—C253—C254—C255	1.1 (6)
C21—C22—C23—O24	−126.5 (11)	C253—C254—C255—C256	−1.3 (6)
C221—C22—C23—O24	107.5 (15)	C254—C255—C256—C251	0.8 (5)
O21—C21—O22—C24	−4.5 (14)	C252—C251—C256—C255	0.0 (5)
C22—C21—O22—C24	−178.5 (7)	N242—C251—C256—C255	−178.7 (3)

### Diethyl (RS)-2-[(4-chlorophenyl)(5-methyl-3-oxo-2-phenyl-2,3-dihydro-1H-pyrazol-4-yl)methyl]propanedioate (II) Hydrogen-bond geometry (Å, º)

**Table:**

*D*—H···*A*	*D*—H	H···*A*	*D*···*A*	*D*—H···*A*
N141—H141···O243	0.83 (3)	1.90 (3)	2.692 (3)	160 (3)
N241—H241···O143^i^	0.85 (3)	1.86 (3)	2.704 (3)	171 (3)
C14—H14*A*···O21^ii^	0.99	2.35	3.322 (11)	166
C132—H132···O13^iii^	0.95	2.58	3.416 (4)	148
C235—H235···*Cg*1	0.95	2.71	3.406 (5)	131
C235—H235···*Cg*2	0.95	2.72	3.444 (7)	133

Symmetry codes: (i) *x*−1/2, −*y*+3/2, *z*−1/2; (ii) *x*+1/2, −*y*+3/2, *z*+1/2; (iii) −*x*+1, −*y*+1, −*z*+1.
